# Antioxidant Capacity Determination in Plants and Plant-Derived Products: A Review

**DOI:** 10.1155/2016/9130976

**Published:** 2016-12-04

**Authors:** Aurelia Magdalena Pisoschi, Aneta Pop, Carmen Cimpeanu, Gabriel Predoi

**Affiliations:** ^1^Faculty of Veterinary Medicine, University of Agronomic Sciences and Veterinary Medicine of Bucharest, 105 Splaiul Independentei, Sector 5, 050097 Bucharest, Romania; ^2^Faculty of Land Reclamation and Environmental Engineering, University of Agronomic Sciences and Veterinary Medicine of Bucharest, 59 Marasti Blvd, Sector 1, 011464 Bucharest, Romania

## Abstract

The present paper aims at reviewing and commenting on the analytical methods applied to antioxidant and antioxidant capacity assessment in plant-derived products. Aspects related to oxidative stress, reactive oxidative species' influence on key biomolecules, and antioxidant benefits and modalities of action are discussed. Also, the oxidant-antioxidant balance is critically discussed. The conventional and nonconventional extraction procedures applied prior to analysis are also presented, as the extraction step is of pivotal importance for isolation and concentration of the compound(s) of interest before analysis. Then, the chromatographic, spectrometric, and electrochemical methods for antioxidant and antioxidant capacity determination in plant-derived products are detailed with respect to their principles, characteristics, and specific applications. Peculiarities related to the matrix characteristics and other factors influencing the method's performances are discussed. Health benefits of plants and derived products are described, as indicated in the original source. Finally, critical and conclusive aspects are given when it comes to the choice of a particular extraction procedure and detection method, which should consider the nature of the sample, prevalent antioxidant/antioxidant class, and the mechanism underlying each technique. Advantages and disadvantages are discussed for each method.

## 1. Introduction

Metabolism implies oxidative processes vital in cell survival. In the course of molecular oxygen stepwise reduction, a series of reactive oxygenated species occur [1–3]. Reactive species may be oxygenated/nitrogenated free radicals defined as chemical species possessing an unpaired electron in the valence shell (superoxide anion radical O_2_^∙−^, hydroxyl HO^∙^, hydroperoxyl HO_2_^∙^, peroxyl ROO^∙^, alkoxyl RO^∙^, nitric oxide NO^∙^, peroxynitrite ONOO^−^, and nitrogen dioxide NO_2_) or neutral molecules (H_2_O_2_ or HClO) [4–7].

Free radicals generated in aerobic metabolism are involved in a series of regulatory processes such as cell proliferation, apoptosis, and gene expression. When generated in excess, free radicals can counteract the defense capability of the antioxidant system, impairing the essential biomolecules in the cell by oxidizing membrane lipids, cell proteins, carbohydrates, DNA, and enzymes. Oxidative stress results in cytotoxic compounds occurrence (malonyl dialdehyde, 4-hydroxynonenal) and alters the oxidant-antioxidant balance (redox homeostasis) that characterizes normal cell functioning [2–4].

With respect to alteration in the protein structure, amino acid oxidation, free radical-induced cleavage, and cross-linking due to reaction with lipid peroxidation products may occur [8]. In nucleic acids, structural alterations imply generation of base-free sites, deletions, oxidation of bases, frame shifts, strand breaks, DNA-protein cross-links, and chromosomal arrangements. The peroxyl radicals and the Fenton-generated OH radicals can induce the oxidation not only of purine and pyrimidine bases but also of the deoxyribose moiety [9, 10]. Regarding influences that involve sugar chemistry, oxygenated free radicals which resulted in early glycation stages have been proven to be contributors to glycoxidative damage: glycolaldehyde that results in the initial stages of nonenzymatic glycosylation is noncyclizable and may undergo tautomerization, yielding enediols that are easily subject to autooxidation. This step is initiated and propagated by superoxide radical. *α*- and *β*-dicarbonyls may also result during this glycolaldehyde autooxidation [11]. Peroxidation of lipids means primarily the attack to the fatty acid's chain by a radical, which abstracts a hydrogen atom from a methylene group, with polyunsaturated fatty acids being the most susceptible to undergo this process. OH^•^, as one of the most active radical species, and HO_2_^∙^ attack lipid substrates (L-H), yielding the corresponding lipid radicals L^•^. The attack on polyunsaturated fatty acids by singlet oxygen can yield lipid peroxides [12, 13].

In recent studies, it has been repeatedly asserted that oxidative stress not only is not limited to free radical-induced damage on biomolecules but also involves perturbation of cellular redox status, which has been described as “a disruption in redox signaling and control”; hence the antioxidant system implies more than mere free radical capture [14–17].

Oxidative stress-induced pathology includes cancer [18, 19], cardiovascular disease [20], neural disorders [21], Alzheimer's disease [22], mild cognitive impairment [23], Parkinson's disease [24], alcohol induced liver disease [25], ulcerative colitis [26], atherosclerosis [27], and aging [28].

The antioxidant action mechanism cannot be understood without describing the model lipid peroxidation in cell membranes or foodstuffs, a radical mechanism that these biomolecules undergo, with initiation, propagation, and chain termination stages, which is promoted by heat, light, and ionizing radiation or by metal ions or metalloproteins [29–31].

 Initiation:
  (1) $$\begin{array}{c} LH+R^{∙}⟶L^{∙}+RH \end{array}$$

LH is the lipid substrate, R^•^ is the initiating oxidizing radical, and L^•^ is the allyl radical endowed with high reactivity.

 Propagation:
  (2) L∙+O2⟶LOO∙LOO∙+LH⟶L∙+LOOH

So, during this step, the lipid peroxyl radicals LOO^•^ act as chain carriers, further oxidizing the lipid substrate and generating lipid hydroperoxides (LOOH), which can decompose into alcohols, aldehydes, alkyl formates, ketones, hydrocarbons, and radicals such as lipid alkoxyl radical LO^•^ [3, 32].

 Branching:
  (3) LOOH⟶LO∙+HO∙2LOOH⟶LOO∙+LO∙+H2O

The decay of lipid hydroperoxides often takes place in the presence of transition metal ions, generating lipid peroxyl and lipid alkoxyl radicals:

(4) LOOH+Mn++H+⟶LO∙+Mn+1++H2OLOOH+Mn+1++OH−⟶LOO∙+Mn++H2O

*Termination* implies the combination of radicals to form nonradical chemical species:

(5) LO∙+LO∙⟶nonradical  productsLOO∙+LOO∙⟶nonradical  productsLO∙+LOO∙⟶nonradical  products

Antioxidants can act as chain breakers, scavenging chain initiating radicals like hydroxyl, alkoxyl, or peroxyl, quenching singlet oxygen, decomposing hydroperoxides, and chelating prooxidative metal ions [13, 33]. Epidemiological studies confirm that the incidence of oxidative stress-related conditions is lowered by the consumption of fruits and vegetables rich in compounds possessing high antioxidant activity [18, 34–37]. Foods containing antioxidants and antioxidant nutrients play an important role in prevention.

Chain breaking antioxidants able to scavenge radical species are called primary antioxidants. Secondary antioxidants are singlet oxygen quenchers, peroxide decomposers that yield nonradical species, oxidative enzyme (e.g., lipoxygenase) inhibitors, UV radiation absorbers, or compounds that act by metal chelating [38–40].

Natural antioxidants constitute the essential part in the cell's defense mechanisms and they can be* endogenous or exogenous*.

*Endogenous* antioxidants can be nonenzymatic, such as glutathione, alpha-lipoic acid, coenzyme Q, ferritin, uric acid, bilirubin, metallothionein, l-carnitine, melatonin, albumin, and antioxidant enzyme cofactors, or enzymatic, such as superoxide dismutase, catalase, glutathione peroxidases, thioredoxins, and peroxiredoxins. Peroxiredoxins regulate cytokine-induced peroxide levels and mediate cell signal transduction [41].

Enzymatic antioxidants at their turn are grouped within the primary and secondary defence systems. The primary defence is formed by three crucial enzymes capable of preventing the occurrence or neutralizing free radicals: glutathione peroxidase, which donates two electrons that reduce peroxides, catalase that decomposes hydrogen peroxide into water and molecular oxygen, and superoxide dismutase that turns superoxide anions into hydrogen peroxide [13, 41]. The secondary enzymatic defense comprises glutathione reductase and glucose-6-phosphate dehydrogenase. Glutathione reductase turns glutathione into its reduced form, thus recycling it. Glucose-6-phosphate reforms reductive NADPH [42, 43]. Although these two enzymes do not directly neutralize free radicals, they promote the endogenous antioxidants' activity [13]. It has been assessed that enzymatic antioxidants act by decomposing free radicals and in this case damaging oxidative species are converted into hydrogen peroxide and water, while nonenzymatic antioxidants are mainly chain breakers. For instance, it has been reported that tocopherol disrupts a radical oxidation chain after five reactions [44].

Apart from the endogenous, enzymatic, and nonenzymatic antioxidants previously discussed, there are also* exogenous*, diet-sourced antioxidants [40, 43], represented by carotenoids, tocopherols, vitamin D, phenolic acids, flavonoids, or ascorbic acid, as well as high-molecular weight metabolites such as tannins. For this second category, the source is represented by foodstuffs, pharmaceuticals, and food supplements. They are important in counteracting the reactive oxygenated species, when the endogenous compounds are not able to ensure thorough protection [40, 43, 45–47].

The intake of antioxidants from diet is always meant to counterpart the organism's antioxidant defense. Enzymic natural antioxidants in food (superoxide dismutase, glutathione peroxidase, and catalase) can be inactivated during processing.

Particularly plant-sourced low-molecular weight antioxidants such as glutathione and ascorbate are synthesized within the chloroplast stroma and the cytosol in the presence of reduced coenzyme molecules (NADPH) acting as the final electron source [48]. These low-molecular weight antioxidants, the cell's redox buffer, are involved in plant growth and development, as they are able to modulate processes from mitosis and cell elongation to senescence and death [49, 50]. Commercial synthetic antioxidants with phenolic structure such as BHA, BHT, and TBHQ are added to foodstuffs to prevent lipid rancidity [51] and the difference in structure transduces itself in antioxidant capacity difference [38].

Although review papers have been previously published on antioxidant activity in plants, the present paper provides a novel way of gathering and also critically and comparatively presenting these aspects. The section devoted to critical and conclusive aspects provides the reader with an original discussion over extraction techniques and their comparison, as well as methods' performances (in a way that has not been systematized until now), with the following aspects concerned: sample, mechanism underlying the method, working parameters, and detection.

## 2. Antioxidant Extraction Procedures

Extraction techniques aim not only at extracting the active biocompounds from the plant sample but also at imparting selectivity and optimizing sensitivity of the applied analytical methodology due to the increase of the concentration of the compound of interest. The biocompound is more easily detected and separated from other matrix components, and the assay becomes independent on the variable matrix characteristics [52].

Classical extraction techniques are based on the extractive potential of various solvents, using heating or mixing. The main shortcomings of conventional extraction are long extraction times, the need for high purity expensive solvents, evaporation of solvents in significant amounts, reduced selectivity, and, finally, the thermal decomposition in the case of thermolabile substances [53]. These problems can be solved by nonconventional extraction techniques that are mainly regarded as “green techniques,” as they use less toxic chemicals, safer solvents, which are characterized by better energy efficiency and minimum by-product amounts [54].

An important goal is represented by high extraction efficiency and efficacy. Efficiency was defined as the yield of extraction, whereas efficacy represents the potential to induce bioactivity and the ability to produce an effect. Therefore, a selection of the most appropriate extraction method is required in each case, as it was proven that various techniques applied on the same plant material employing the same solvent can lead to different extraction efficiencies. Moreover, it has been confirmed that the most convenient method in this regard requires standardization to attain reproducibility [55].

### 2.1. Conventional Techniques

*Soxhlet extraction* was first applied only for lipid extraction, but its use has been extended for extracting active principles. The solvent is heated, vaporized, and condensed and extracts the interest compound(s) by contact with the sample-containing thimble. When the solvent in the extraction chamber reaches the overflow level, the solution in the thimble-holder is aspirated by a siphon and returns in the distillation flask. Significant extraction yields can be reached, with a small solvent amount. It can be applied in batch at small scale, but it can be converted into a continuous extraction set-up on medium or large scale [56].

*Maceration* is applied to obtain essential oils and bioactive compounds. The plant material is ground to improve the surface area. The solvent is then added and allowed to stand at ambient temperature for several days, and the mixture is subject to frequent stirring until dissolution. The damped material is then pressed and then the liquid is purified by filtration or decantation [54, 56].

*Hydrodistillation* (as water, water/steam, and direct steam distillation) is applied to the extraction of bioactive compounds and essential oils from plants, generally prior to dehydration, and does not imply the use of organic solvents [57]. Hot water and steam isolate the bioactive compounds from the plant tissue. Consequently, cool water condenses the vapor mix of water and oil. The condensed mixture reaches the separator, where oil and biocompounds are isolated from water [58]. Hydrodistillation involves three main steps, hydrodiffusion, hydrolysis, and thermal decomposition, with the risk being represented by the decay of thermolabile substances [54, 56].

*Infusions* are prepared by shortly macerating the raw plant material with either cold or boiling water. It is often mentioned that concentrated infusions are the result of a modified percolation or maceration procedure [56].

*Percolation* is a recognized procedure applied for the preparation of tinctures and fluid extracts and makes use of a cone-shaped vessel opened at both ends (percolator). The solid material is moistened with an adequate amount of the appropriate solvent (menstruum) and left for about 4 h. Solvent amount is necessary, until the percolate represents about three-quarters of the quantity corresponding to the final product. The marc is then pressed and the eliminated liquid is added to the percolate. Solvent is again added to get the required volume, and the liquid mixture is clarified by filtration or by decanting [56].

In the* decoction* process, the crude plant material is subject to boiling in an appropriate water amount, for a well-defined period, followed by cooling and then straining or filtering. This approach is adequate for the extraction of hydrosoluble, thermostable components, being popular for obtaining Ayurvedic extracts [56].

*Cold pressing or expression* consists in pressing or grinding fruits or seeds by using a press. Oil release is possible due to crushing or breaking of essential oil glands in the peel. Olive, peanut, sunflower, and citrus oils are obtained through cold pressing, which results in preserving flavor, aroma, and nutritional value.

*Aqueous Alcoholic Extraction by Fermentation*. The formed ethanol enables extraction of the active principles from the material and also contributes to preserving the product's qualities. In Ayurveda, this method is not standardized, but, with progresses in the fermentation technology, standardization would be of use for obtaining herbal drug extracts [56].

*Vortex* apparatus is commonly used to mix the interest plant sample with the dilutant. It is applied for dissolution, namely, in aqueous environment and polar solvents, of samples of plants to yield a fluid and homogeneous solution subject to analysis. As in the case of other techniques like shaking or sonication, it can be followed by centrifugation, with use of the supernatant.

### 2.2. Nonconventional (Modern) Techniques

*Supercritical Fluid Extraction (SFE).* Critical point was defined as the temperature and pressure above which distinction between gas and liquid phases does not exist [59]. In supercritical state, gas and liquid properties are not individualized, and supercritical fluid properties are tunable by temperature and pressure modification. Supercritical fluids (SCFs) possess both gas-like properties (diffusion, viscosity, and surface tension) and liquid-like density and solvation power [60]. The advantages are constituted by reduction of extraction time when compared to conventional methods, complete extraction by repeatable refluxes, better selectivity in comparison to common liquid solvents due to solvation power, enhanced transport properties exhibited near the critical point, and hence high extraction yields [55]. CO_2_ use does not imply high costs. It operates at room temperature, so it is adequate for thermosensitive compounds; smaller samples can be extracted compared with conventional solvent extraction. It is characterized by facility of coupling with chromatographic procedures and reutilization of SCF [54, 56]. Disadvantages may be represented by polarity limitations of carbon dioxide, which can be minimized by the use of organic solvents, or inert gases (Ar) [56].

*Solid Phase Microextraction (SPME)*. SPME employs a sorbent, which usually coats the surface of small fibers, for the isolation and concentration of target compounds from the sample and is applied to quantitative assay of analytes (essentially flavor compounds) in aqueous or gaseous phase.

*Microwave-Assisted Extraction (MAE)*. Microwaves interact with the dipoles of polar and polarizable matrixes [61, 62]. As the forces of electric and magnetic field components swiftly modify their orientation, polar molecules also adopt orientation in the changing field direction, and heat is generated. So, ionic conduction and dipole rotation are the mechanisms underlying the conversion of electromagnetic energy to heat [54, 63]. The components of the sample absorb microwave energy in conformity with their dielectric constants [64]. When the plant material is found in a solvent transparent to microwaves, the elevated vapour pressure causes rupture of the cell wall of the substrate and frees the content into solvent [55]. Separation of solute molecules from the sample matrix at increased temperature and pressure is followed by diffusion of solvent molecules across the sample matrix and transfer of solute molecules from the sample matrix to the solvent. Microwave-assisted extraction is characterized by rapid heating to reach the temperature required for extracting bioactive principles [65], enhanced extraction yields, very good recovery and selectivity, and minimum equipment size and solvent use [54, 66].

*Ultrasound-Assisted Extraction (UAE)*. Ultrasound waves with frequencies comprised between 20 kHz and 100 MHz induce compression and expansion as they pass through the extractable plant matrix, producing cavitation. The energy produced can promote the conversion of kinetic energy into thermal one, inducing heating of the bubble contents. In solid plant samples, ultrasounds enable compound leaching from the plant materials [67]. The mechanism implies wave diffusion across the cell wall and rinsing of the cell's content after breaking the walls [68]. The physical, chemical, and mechanical forces induced by the collapse of bubbles result in the disruption of membranes to enable the release of extractable compounds and to facilitate penetration of the solvent into cell material [69, 70]. Rapidity, intensified mass transfer, low solvent amounts, high extraction yields and throughput, and reduced temperature gradients characterize this technique [54]. Nevertheless, high ultrasound energy may result in cell membrane impairment due to free radical generation, but the deletions can be resealed by aggregation of lipid vesicles [71].

*Pulsed-Electric Field (PEF) Extraction*. Living cells are suspended in an electric field, and an applied potential crosses the membrane. The electric potential induces molecule separation according to the molecular charge. At values greater than 1 V for the transmembrane potential, the electrostatic repulsion between the charged molecules results in pore generation in the membrane and produces dramatic permeability increase, and yield is optimized [54, 72]. The efficacy of the pulsed-electric field extraction depends on field strength, energy input, pulse number, temperature, and matrix characteristics [73]. Pulsed-electric field extraction is also appliable as pretreatment before carrying out traditional extraction [74]. It can be employed before grape skins maceration, minimizing maceration time and imparting stability to anthocyanins and polyphenols [75].

*Enzymatic Treatment*. Enzymes used are cellulase, *α*-amylase, and pectinase, which act by breaking the cellular wall, with subsequent hydrolysis of the structural polysaccharides and lipids [76, 77]. Enzyme-assisted aqueous extraction and enzyme-assisted cold pressing are the main techniques applied [78]. Enzyme amount, particle size of the material, solid to moisture ratio, and hydrolysis time influence the performances [79]. Enzyme-assisted cold pressing is the most proper for extracting biocompounds from oilseeds as nontoxic procedure, which does not involve flammable liquids. The oils extracted are richer in fatty acids and phosphorus than hexane-extracted ones [80]. The enzyme-assisted aqueous extraction is environmental-friendly [81]. In enzyme-assisted cold pressing, biocatalysts hydrolyse the seed cell wall, because the polysaccharide-protein colloid is not present, as happens in the enzyme-assisted aqueous extraction [82].

*Pressurized Liquid Extraction (PLE)*. PLE implies exerting an elevated pressure to the remaining liquid solvent above the boiling point. High pressure values favor the extraction process, which is easily prone to automation. Pressurized liquid extraction benefits much shorter extraction times and lower solvent requirements, when compared to conventional Soxhlet extraction. At elevated temperatures and pressures, the extraction performances are improved by the increased analyte solubility and mass transfer rate, as well as by the diminished viscosity and low surface tension of solvents [54, 83].

## 3. Analytical Methods Applied to Antioxidant Content and Antioxidant Capacity Assessment in Plant Extracts: Classification and Principles

The investigation of performant analytical methods aiming to assess the antioxidant capacity in plants and plant extracts remains a constant goal and a series of classifications have been proposed. Antioxidant measurement techniques were classified as methods based on the inhibition of low-density lipoprotein oxidation estimation and the ones relying on the quantification of the free radical scavenging capacity [84].

Considering the mechanism underlying the antioxidant–oxidant reaction, the methods were also divided in hydrogen atom transfer (HAT) and single electron transfer (SET) techniques. HAT-based methods measure the capacity of an antioxidant to trap free radicals by hydrogen donation, while SET methods rely on the one electron transfer reductive ability of an antioxidant compound versus a radical species [85]. ORAC, TRAP, and chemiluminescence are hydrogen atom transfer-based methods, whereas FRAP and CUPRAC are single electron transfer methods [85]. DPPH and TEAC methods were regarded as methods using both hydrogen and single electron transfer, as the radicals in these cases can be scavenged by either electron reduction or radical quenching that involves hydrogen transfer [85, 86]. DPPH scavenging, TEAC assay, ferric reducing antioxidant power, OH^•^ scavenging, the phosphomolybdenum method, and beta-carotene linoleate bleaching are applied* in vitro*, while the lipid peroxidase, catalase, and glutathione peroxidase activity assays are techniques used* in vivo* [18]. The analytical response is also recorded as per reference to a standard antioxidant: Trolox, gallic acid, ascorbic acid, caffeic acid, and so forth.

The main chemical processes underlying antioxidant activity assay (a–d) and lipid oxidation status evaluation (e) are detailed in Table 1. The latter are presented, as they can constitute the basis for antioxidant screening: the assays can be performed by following the prevention of peroxidation products generation in the presence of antioxidants, measured against a control. The determinations may involve hydroperoxide, conjugated diene, or thiobarbituric acid reactive substances assay. The antioxidant effect is expressed as percent of lipid peroxidation inhibition. The group of techniques involving low-density lipoprotein peroxidation inhibition by antioxidants is also classified as belonging to HAT methods, as the reaction between the antioxidant and the peroxyl radicals (such as AAPH-initiated) involves hydrogen transfer.

In Table 2, the methods are classified following the detection mode, with principle description for each technique.

## 4. Significant Analytical Applications to Plant and Plant Extracts

### 4.1. Chromatography

#### 4.1.1. Planar Techniques

Thin layer chromatograms of the methanolic extract of* Bergia suffruticosa* (used as bone and sore healer) proved antiradical activity by bleaching DPPH^•^. This free radical scavenging activity was assigned to the high tannin and phenolic amounts [90]. A recently developed TLC–DPPH^•^ assay allowed for the swift detection of the antioxidant potential of nine out of ten tested polyphenols (except for apigenin 7-O-glucoside), present in five analysed plant species:* Hypericum perforatum L.*,* Matricaria recutita L.*,* Achillea millefolium L.*,* Thymus vulgaris L.*, and* Salvia officinalis L*. By LC–MS, the presence of compounds previously identified by TLC was confirmed. Four other compounds (caffeic acid and apigenin in St. John wort and apigenin and apigenin 7-O-glucoside in sage) have been identified. Their presence was not revealed by TLC and it has been stated that their low level in the plant samples could be the reason [91].

*Sonneratia caseolaris* (astringent and antiseptic) extracts were tested for their antioxidant composition: column chromatography with a Diaion HP-20 column and successive elution with methanol and acetone was first applied. The chlorophyll-free eluate was separated into 5 fractions by C18 column chromatography, with methanol and acetone for elution. The methanol-eluted fraction containing DPPH positive spots was then applied to a silica gel presqualene column with n-hexane-acetone–methanol eluents, resulting in eight fractions. The first compound was obtained after precipitation from the fraction corresponding to the n-hexane-acetone 1 : 1 eluate. One acetone-eluted fraction also yielded a precipitate, which after washing with methanol resulted in the second compound. The structures of the isolated compounds were assessed by one-dimensional and two-dimensional NMR and mass spectroscopy. Moreover, both showed positive (discolored) spots with a reddish purple background on the thin layer chromatogram, using a 0.02% (w/v) methanolic solution of DPPH as spray reagent. Luteolin and luteolin-7-O-*β*-glucoside were identified as the two bioactive antioxidant and anti-inflammatory compounds [92].

High performance thin layer chromatography combined with densitometry was applied for caffeic acid quantitation in* Plantago lanceolata*. The best eluent composition was determined: in first step of development, the mobile phase contained hexane, diisopropyl ether, and formic acid 90% (6.0 : 4.0 : 0.5) v/v. In the second and third steps, a mixture of hexane, diisopropyl ether, dichloromethane, formic acid 90%, and propan-2-ol (6.0 : 4.0 : 2.0 : 1.0 : 0.1) v/v was employed. The application of this HPTLC technique with area measurements at 320 nm led to a caffeic acid amount equal to 99.3 *μ*g/g of dried plant, with RSD of 3.19% [93].

HPTLC [94] was also used for the screening and quantitation of phytochemicals present in* Scoparia dulcis*, known for many health benefits [95] (see Table 3). After application of the anisaldehyde-sulphuric acid visualization reagent, the spotted plate was exposed to UV radiation (254 and 366 nm) and multicolored bands at various intensities were noticed. On the TLC plates, the presence of phenolics (flavonoids) and terpenoids has been revealed [94].

The antioxidant capacity of essential oils obtained from the seed and whole plant of* Coriandrum sativum* was assessed, and HPTLC was applied to assess significant phytomarkers. The* in vitro* determined antioxidant capacity was greater than the one corresponding to various extracts of this Ayurvedic plant. The chromatographic profile showed linalool and geranyl acetate as main phytoconstituents of the analysed samples. The HPTLC system was based on a TLC scanner, an autosampler connected to a nitrogen cylinder, a UV scanner, and visualizer. The limits of detection and quantification were obtained as 0.4 and 1.2 ng/mL for linalool and 0.6 and 1.4 ng/mL for geranyl acetate, revealing sensitivity. The precision was proven by the result of minimum six replicate analyses, with a coefficient of variability of 0.07% [96].

#### 4.1.2. Column Techniques

*(1) Gas Chromatography*. The composition of various extracts of* Merremia borneensis* was assessed by GC-MS, showing the presence of flavonoids, terpenoids, alkaloids, and glycosides in the analysed organic crude extracts [97]. The qualitative analysis of bioactive compounds present in* Datura metel* was performed in crude extracts by GC/MS, revealing abundancy of high-molecular weight components such as polyphenols, flavonoids, triterpenoids, and hydrocarbons. The phenolic level was expressed as gallic acid equivalents, with chloroform having the best extractive potential, followed by methanol, butanol, ethyl acetate, and hexane. It has been concluded that the chloroform crude extract had the highest phenolics amount and its potential as antibiotic has been stated [98].

Essential oils from the aerial parts of* Ajuga bracteosa *and* Lavandula dentata* obtained by hydrodistillation were analysed by GC and GC/MS. 47 and 48 biocomponents were identified for the two analysed plants, respectively. The oils contained high amounts of oxygenated monoterpenes (34 to 51%). Borneol (20.8%) and hexadecanoic acid (16.0%) were the major compounds present in the oil of* A. bracteosa, which also contained *aliphatic acids (30.3%). Camphor (12.4%),* trans*-pinocarveol (7.5%), and *β*-eudesmol (7.1%) were prevalent in* Lavandula dentata oil*. The antioxidant activity of the oil extracts was confirmed by DPPH^•^ scavenging assay [99].

*(2) Liquid Chromatography*. The rapid and resolution-high determination of six bioactive flavonoids present in the pericarp of* Citri reticulata* has been performed by liquid chromatography/electrospray ionization coupled with mass spectrometry. The chromatographic system used a C18 column and a 0.1% formic acid/acetonitrile mobile phase with a gradient elution. Naringin, hesperidin, nobiletin, 3,5,6,7,8,3′,4′-heptamethoxyflavone, tangeritin, and 5-hydroxy-6,7,8,3′,4′-pentamethoxyflavone were assessed by the above-mentioned chromatographic technique and were also investigated for their antiproliferative activities by Cell Counting Kit-8 Assay. In the cultivars analysed, hesperidin presented the highest content, ranging from 50.137 to 100.525 mg/g. The levels of nobiletin, tangeritin, and 5-hydroxy-6,7,8,3′,4′-pentamethoxyflavone were higher in the peel of* Citrus reticulata* “Chachi” than in other cultivars. With respect to the antiproliferative activity against A549 and HepG2 cells, 5-hydroxy-6,7,8,3′,4′-pentamethoxyflavone has been proven to be the most effective [100].

Chromatography followed by electrochemical detection proved its viability in the assessment of onion (*Allium cepa*), parsley (*Petroselinum crispum*) roots and leaves, celery (*Apium graveolens*) roots, and leaves of dill (*Anethum graveolens*) extracts, relying on the antioxidant compounds' specific oxidation. It has been confirmed that the method is characterized by sensitivity and simplicity of detection, since no additional instrumentation (reagent pump or secondary detector) is necessary. In comparison to the results obtained using reversed-phase chromatographic separation with online postcolumn DPPH scavenging detection, HPLC-ED provided much richer chromatographic profiling of celery leaves extracts. At elevated electrooxidation potential values higher than 700 mV, compounds that are electroactive contribute to HPLC-ED detection but are missed in the postcolumn DPPH scavenging [101].

The HPLC chromatograms of* Carissa opaca* various fractions proved the presence of orientin, isoquercetin, myricetin, and apigenin endowed with antioxidant activity. The antibacterial, antitumoral, and anticarcinogenic potential of these flavonoid-rich fractions of* Carissa opaca* has also been confirmed in this study [102].

Eleven Algerian medicinal plants were subject to analysis for their antioxidant capacity and phenolic profile. The HPLC results revealed that the hydroxycinnamic acid derivatives were the predominant phenolics of the extracts endowed with best antioxidant activity (*Anthemis arvensis* and* Artemisia campestris*). Nevertheless, it was stated that in this case the correlation between the antioxidant activity of analysed extracts and their phenolic composition is very difficult to be described by statistical tools. It was assumed that this difficulty may result not only from the fact that total phenolics do not include all the antioxidants but also from the synergism and structure interaction among the antioxidants, which does not always involve concentration influence. For instance, samples such as* Artemisia arborescens* and* Oudneya africana*, with close concentration values of total phenolics, exhibited varying antioxidant activity. On the whole, the antioxidant activity and flavonoids concentration did not correlate significantly in comparison to hydroxycinnamic acids and hydroxybenzoic acids.* Artemisia campestris* was assessed as the most powerful inhibitor of radical-induced red blood cells hemolysis, more active than caffeic acid, more than three times more active than ascorbic acid, and two times more active than *α*-tocopherol. The UV spectra were obtained in the range of 220–600 nm and the amounts of phenolics in the extracts were assessed from the calibration curves developed at the absorption maxima of each phenolic class [103].

Methanolic extracts of the leaves of* Rosmarinus officinalis* were assessed by HPLC for their radical scavenging antioxidant activities. The identified compounds, namely, carnosol, carnosic acid, and rosmarinic acid, varied as depending on the geographical regions and season. The chromatographic system involved a C18 column and a mobile phase composed of methanol and acetic acid/acetonitrile, with gradient elution. The highest content of carnosic acid was obtained in the samples harvested from Mersin; the highest rosmarinic acid level was assigned to Canakkale-originating samples (14.0–30.4 mg/g). For all extracts, the carnosol content ranged from 5.4 to 25.5 mg/g, and the carnosic acid level ranged from 3.8 to 115.8 mg/g [104].

The phenolic ingredients in samples of 24 cereal grains were analysed by HPLC, relying on the peak area of maximum absorption wavelength. The chromatographic setup was comprised of a C18 column, a mobile phase with an elution gradient between solution A (acetic acid-water and methanol) and solution B (methanol and acetic acid-water solution), and a photodiode array detector. Gallic acid, kaempferol, quercetin, galangin, and cyanidin 3-glucoside were found in high amounts in these cereals [105].

HPLC was also applied along with LC-MS for the estimation of polyphenolic compounds from bitter cumin. The amount of phenolic compounds (*μ*g/g dry weight) was estimated by comparing the peak areas (at 254 nm) of the samples with that of standards, proving the prevalence of caffeic acid: 500.0 *μ*g/g dry weight [106].

The profile and quantitative analysis of compounds present in* Lycium* species was performed using HPLC with diode array detection:* p*-coumaric acid, chlorogenic acid, and rutin were identified by their retention times and UV spectra versus those of the standards. Other benzoic and hydroxycinnamic acids, flavonoids, and anthocyanin derivatives were identified by UV spectra and quantified by using gallic acid,* p*-coumaric acid, rutin, and cyanidin-3-glycoside, respectively, as standards. Phenolic acid derivatives confirmed their prevalence and presence in the highest amounts in all analysed extracts. Butanolic extracts of* Lycium barbarum* and* Lycium ruthenicum* were characterized by the highest level of benzoic and hydroxycinnamic acid derivatives, which was in accordance with the most enhanced antiradical activity of these extracts [107].

HPLC with diode array detection and ion trap MS was applied to assess dose response and metabolism of anthocyanins present in strawberry. Pelargonidin 3-glucoside was the main anthocyanin present in strawberry, and this anthocyanin and three of its metabolites (detected as monoglucuronides) were excreted and assessed in urine after ingestion. One prevalent monoglucuronide form was detected in urine in masses 10-fold higher than the other two monoglucuronide forms. It was assessed that anthocyanins from strawberries present a linear dose response over ranges of 15–60 mmol. The 24 h urinary recoveries were much more elevated than those reported for most of the other anthocyanins and it has been concluded that pelargonidin-based anthocyanins may be more efficiently absorbed than other anthocyanins [108].

18 phenolic compounds have been analysed by HPLC-MS in harvested and commercial 50% methanolic extracts of* Ocimum basilicum*. In the extracts obtained from harvested samples, rutin (665.052 mg/100 g dried plant) and caftaric acid (1595.322 mg/100 g dried plant) were determined in the largest amount. Commercial samples contained hydroxycinnamic acid derivates, dihydroxybenzoic acid, flavonols, and flavonoid glycosides [109].

The determination of rosmarinic acid content of* Salvia maxima *and* Salvia verde* was carried out by HPLC. Methanol was employed for the extraction of the* Salvia* samples; then filtration (on a 0.45 mm PTFE filter) was performed before injection in the LC-DAD-ESI/MS setup. The mobile phase was comprised of 0.1% (v/v) formic acid and acetonitrile, with the application of linear gradient. The content of phenolics in the analysed samples was assessed through interpolation of the peak area using the calibration curve developed per reference to the rosmarinic acid peak and retention time. The results obtained, as rosmarinic acid equivalent content, ranged from 103 ± 2 *µ*g/g fresh material for* S. maxima* to 174 ± 2 *µ*g/g fresh material for* Salvia verde*, with a limit of detection of 3.4 × 10^−7^ mol L^−1^ [110].

The application of a series of chromatographic techniques (HPLC-DAD, LC-MS/MS, and GC-MS) led to the successful detection of antioxidant purine alkaloids (caffeine, theobromine, and theophylline) and indole alkaloids (harmine, harmane, harmol, yohimbine, brucine, and strychnine) in* Andrographis paniculata* and in dietary supplements containing this plant. This Ayurveda plant is used for healing purposes (see Table 2), hence the interest in structure and potential toxicity elucidation. Purine and indole alkaloids assessment by HPLC-DAD, LC-MS/MS, and GC-MS showed lower concentration of these components in roots of 50.71 ± 0.36 mg/g d.m. in comparison to the leaves of 78.71 ± 0.48 mg/g d.m. In addition, three bioactive diterpenoids were determined by HPLC-DAD and GC-MS methods with good selectivity, accuracy (recovery > 91.5%), and precision (RSD < 5.0%) [111].

The analysis of phenolic synthetic antioxidants BHA, BHT, and TBHQ in edible oils was carried out by HPLC with UV-VIS detection at 280 nm on the basis of peak area ratios. The mobile phase was composed of methanol and 0.01 mol L^−1^ monosodium phosphate, with gradient elution. BHA content ranged between 20.1 *μ*g g^−1^ in rapeseed oil and 55.9 *μ*g g^−1^ in sesame oil. BHT was only found in blend oil at a level of 21.4 *μ*g g^−1^. TBHQ amount ranged between 25.4 *μ*g g^−1^ in rapeseed oil and 47.2 *μ*g g^−1^ in corn oil [112].

A number of 19 phenolic compounds were determined by HPLC, during the ripening of cumin seeds. The phenolic compounds were analysed by* Reversed*-*Phase* High Performance* Liquid Chromatography* with an UV-VIS multiwavelength detection. The separation was performed on a Hypersil ODS C18 column at ambient temperature. The mobile phase comprised acetonitrile and water with 0.2% H_2_SO_4_. The flow rate was established at 0.5 mL/min and gradient elution was applied. Rosmarinic acid was the main phenolic acid found in the unripe seeds. Then, p-coumaric acid was confirmed as the prevalent phenolic in half ripe and full ripe seeds [113]. HPLC analysis of* Artemisia capillaris* extracts proved that the main compounds imparting antioxidant capacity were chlorogenic acid, 3,5-dicaffeoylquinic acid, and 3,4-dicaffeoylquinic acid [114].

**Figure 1:**
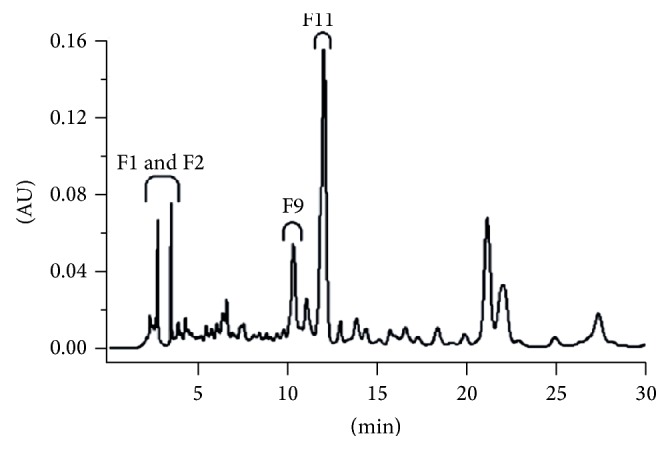
HPLC chromatogram (at 330 nm) of the methanolic extract of* Bambusa*, with illustration of the antioxidant fractions F1, F2, F9, and F11 [87].

The HPLC profile of methanolic extracts of* Spathodea campanulata* revealed antioxidant potential of this traditionally used plant against malaria and inflammation due to the presence of bioactive compounds such as verminoside (10.33%) and 1-O-(E)-caffeoyl-beta-gentiobiose (6.58%) [115]. Glucosinolates from broccoli were analysed by HPLC after enzymatic desulfation. The HPLC system included a Spherisorb ODS-2 column, and the water/acetonitrile mixture was used for gradient elution of samples. Glucoraphanin, precursor of the most active antioxidant glucosinolate found in broccoli, was assessed as the prevalent compound: 14.06 to 24.17 *µ*mol/g [116]. HPLC chromatographic assay of the methanolic extract of* Bambusa textilis *McClure indicated active antiradical fractions, as presented in Figure 1 [87].

### 4.2. Spectrometric Techniques

#### 4.2.1. Studies Based on Nonenzyme Assays

The antioxidant activity of* Acacia confusa* bark extracts was determined by free radical scavenging against DPPH. The total phenolic content was assessed according to the Folin-Ciocalteu method, using gallic acid as a standard. The scavenging activity exhibited against the DPPH free radical diminished in the following order: 3,4,5-trihydroxybenzoic acid = 3,4-dihydroxybenzoic acid = 3,4-dihydroxybenzoic acid ethyl ester > 4-hydroxy-3-methoxybenzoic acid > 3-hydroxy-4-methoxybenzoic acid > 4-hydroxybenzoic acid = benzoic acid. It has been stipulated that this trend is due to the presence of catechol moieties in 3,4,5-trihydroxybenzoic acid, 3,4-dihydroxybenzoic acid, and 3,4-dihydroxybenzoic acid ethyl ester, which impart antioxidant activity [207].

Fruits of* Lycium* species were subject to sequential extraction with petroleum ether, ethyl acetate, methanol, n-butanol, and water in a Soxhlet extractor. All the extracts were analysed for their scavenging potential towards the free DPPH^•^ radical by* in vitro* method. The composition of each extract was also studied for the Folin-Ciocalteu reactive species. It was stressed out that the butanol extracts of both species (*Lycium barbarum* and* Lycium ruthenicum*) were endowed with the highest scavenging potential (smallest IC_50_). A linear relationship (correlation) was established between the total phenol content (Folin-Ciocalteu assay) and the radical scavenging potential [107].

A research dedicated to the antioxidant composition investigation and antioxidant capacity determination in* Ocimum basilicum* showed that the scavenging effect against the DPPH radical was proportional to the phenolic content, to which flavonoids and caffeic acid derivatives contribute. The DPPH scavenging activity proper to harvested samples (26.55 and 22.43%, resp.) was greater than the one obtained for commercial ones (12.05 and 11.24%), considering the one of BHT as 94.77% [109].

Six spice plant samples, namely, onion (*Allium cepa*), parsley (*Petroselinum crispum*) roots and leaves, celery (*Apium graveolens*) roots and leaves, and leaves of dill (*Anethum graveolens*), were subject to analysis for the total phenolic amount and the antioxidant activity assessed by DPPH scavenging. The celery leaves exhibited the highest total phenolic content, namely, 1637.1 mg gallic acid equivalents/100 g, and the highest radical scavenging activity against DPPH [101].

The antioxidant properties of methanol extracts of 15 broccoli samples were estimated by DPPH^•^ and OH^•^ radical inhibition. These activities ranged from 1.49 *µ*mol Trolox/g DW to 3.34 *µ*mol Trolox/g DW. The sample endowed with the highest DPPH^•^ radical scavenging activity also possessed the highest phenolic and glucosinolate contents, including glucoraphanin [116].

In another study, the antioxidant activity of* Clusia fluminensis* extracts was assessed, exploiting the scavenging of the stable free radical DPPH. The flavones and flavonols content was also determined in order to test the potential correlation with the antioxidant capacity. No significant differences were revealed between the total flavonoid contents of* Clusia fluminensis* in acetone and methanol extracts, respectively. The acetone extract was endowed with the highest antioxidant activity (with almost 2 times smaller EC_50_ value than the one proper to methanol extract) and highest flavonoid level. Hence, it has been asserted that acetone is an efficient solvent for antioxidant extraction. It has been also suggested that the substances with best antioxidant activity in Clusiaceae fruits possess intermediate polarity [176].

The antioxidant activity of 52 wine samples was assessed spectrophotometrically and expressed as the amount of wine able to engender 50% decolorization of the DPPH radical solution per reference to the control (EC_50_). The obtained average values of EC_50_ were 20.1 *μ*L for red and 98.4 *μ*L for white dry wines. The highest EC_50_ of red dry wines, 26.9 *μ*L (illustrating the lowest antioxidant capacity), was inferior to the one proper to white wines with the most reduced antioxidant capacity, 56.4 *μ*L. It was inferred that, regarding DPPH radical scavenging, red wines are around 5 times stronger than white wines, despite the absence of statistically significant differences between the grape varieties studied, as well as among different wine regions [210].

The total phenolic amount and antioxidant potential expressed by the IC_50_ values (concentration causing a 50% DPPH inhibition) were assessed in the seeds of cumin at different ripening stages. At full ripening stage, for which the highest level of total phenolics was determined (17.74 and 25.15 mg GAE/g DW), the antioxidant capacity also attained its peak, with the smallest values of IC_50_, 6.24 and 42.16 *μ*g/mL, respectively, for maceration and Soxhlet methods applied for extraction [113].

Another study was performed to assess the antioxidant and antimicrobial potential of methanol (100 and 80%) aqueous extracts of pumelo fruits albedo (*Citrus grandis* Osbeck). The antioxidant and antibacterial activity of both crude extracts and isolated compounds were determined using DPPH scavenging and paper disc diffusion method. The 100% methanol extract was steeped in water at different pH values and subject to partitioning with ethyl acetate yielding basic, acidic, neutral, and phenolic fractions. The neutral fraction revealed the highest antioxidant potential and antibacterial efficacy [191].

The antioxidant activities of* Artemisia capillaris* extracts in different organic solvents (*n*-hexane, ethyl acetate, acetone, and methanol) were tested. Methanol extracts of* Artemisia capillaris herba* possessed the highest phenolic content and were endowed with the strongest antioxidant power, when compared to the other solvent extracts; namely, the scavenging potential of three extracts exhibited against the DPPH radical varied as follows: ethyl acetate extracts < acetone extracts < methanol extracts, corresponding to the inhibition percentages of 35.2, 57.1 and 91.1% at a dose of 200 ppm, respectively. The scavenging potential of 100 ppm methanol extract (90.8%) equaled the one proper to 100 ppm BHT (90.5%) and was very close to the one of *α*-tocopherol (92.3%). The methanolic extract also exhibited the strongest antioxidant activity in the *β*-carotene bleaching system [114].

**Figure 2:**
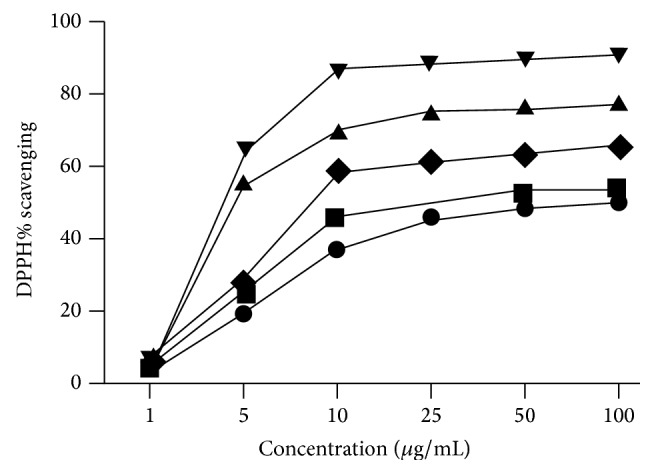
DPPH radical scavenging activity of* Sonchus asper* extracts in different solvents at various concentrations. Each value stands for the mean ± SD (*n* = 3) of hexan, ethyl acetate, chloroform, and methanol crude extracts of the whole plant and ascorbic acid [88].

The antioxidant potential of nonpolar (hexane, ethyl acetate, and chloroform) and polar (methanol)* Sonchus asper* crude extracts was assessed by DPPH radical scavenging. Methanol extract showed the highest scavenging potential (smallest IC_50_) followed by chloroform, ethyl acetate, and hexane extracts, as revealed in Figure 2 [88].

The antiradicalic activity against DPPH and the superoxide anion scavenging activity (in a riboflavin-light-nitro blue tetrazolium chloride system) were determined in the case of methanolic extracts of* Bergia suffruticosa*, bone, and sore healer. The whole plant extracts proved dose-increasing scavenging activities versus DPPH^•^ and superoxide, with EC_50_ values of 13.1 *µ*g and 139.4 *µ*g, respectively. The Fe^3+^ to Fe^2+^ reducing ability was also proven to be dose-dependent, reaching a maximum for 300 *µ*g extract [90].

In another paper, the antioxidant profiling and antioxidant activities of dried fruits have been assessed, namely, prunes, apricots, raisins, and figs [183]. The highest concentration of carotenoids was present in apricots and figs (10.7 and 10.8 mg *β* carotene equivalents/100 g). Raisins possessed the highest total phenolic concentration (1.18 g gallic acid equivalents/100 g) and proanthocyanidins (17.53 mg cyanidin equivalents/100 g). Figs presented the highest flavonoid (105.6 mg quercetin equivalents/100 g) and anthocyanin (5.9 mg/100 g) amount. The antioxidant activities were also assessed. The apricot aqueous extract had the best reducing power, Agen prune presented the highest antioxidant activity furnished by the phosphomolybdenum method, and the raisin extract in ethanol showed the best DPPH^•^ quenching capacity [183].

A study based on ABTS scavenging ability proves that rosemary extract exhibits an antioxidant capacity equivalent to the one of BHA and superior to the one proven by BHT. The* chile ancho* extract exhibits a lower antioxidant capacity when compared to rosemary and both BHT and BHA. In the case of rosemary, due to the preponderance of ethanol in the extraction mixture, the polyphenol amount increases, being the most elevated for an ethanol : water ratio of 75 : 25. The rosemary extract is rich in rosmarinic acid, carnosic acid, and carnosol. In the case of* chile ancho*, the maximum extracted polyphenol amount is obtained for an ethanol : water ratio of 50 : 50. The* chile ancho* extract contains flavonoids (luteolin, quercetin), carotenoids, ascorbic acid, and capsaicinoids [178].

The phenolic content and antioxidant activity of* Vitis vinifera* extracts were followed under different storage conditions. The total phenolic content was determined by Folin-Ciocalteu method and the antioxidant capacity by the scavenging ability versus the ABTS cation radical. The extract proved stable for up to one year at storage in darkness as a hydroalcoholic solution at 4°C or as a freeze-dried powder at 25°C. The total phenolic content was found constant at different pH values (3.0, 5.0, 7.0, and 9.0) for up to 400 days, while the antioxidant capacity diminished at pH values greater than 5.0. The thermal treatment (at 121°C for 15 minutes, at different pH values) neither decreased nor increased the ABTS radical cation-scavenging activity. Nevertheless, it was found that the total phenolic content increased after heating at all pH values tested [181].

Another study was dedicated to the assessment of the antioxidant potency of phenolic substances from wild Algerian medicinal plants by chemical and biological methods.* Anthemis arvensis* and* Artemisia campestris* had the highest phenolics amounts (115.2 and 103.4 mg/g DW, resp.) and the most enhanced antioxidant power assessed by ABTS^•+^ decolorization (0.726 and 0.573 mmol TEAC/g DW, resp.). They also promoted an enhanced delay of free radical-induced red blood cells hemolysis, even when compared to caffeic acid, the reference antioxidant endowed with the most effective inhibition capacity [103].

Both lipophilic and hydrophilic components of 24 cereal grains from China were spectrophotometrically assessed. For water-soluble fractions of the analysed grains, the FRAP values varied from 0.87 ± 0.08 to 114.69 ± 2.15 *µ*mol Fe(II)/g DW. Black rice exhibited the highest FRAP value, followed by organic black rice, purple rice, and organic black millet. With respect to the fat-soluble fractions, the FRAP values ranged between 4.27 ± 0.19 and 21.91 ± 1.27 *µ*mol Fe(II)/g, with red rice, buckwheat, organic black rice, and brown rice exhibiting the most elevated FRAP values. The TEAC values (relying on the ability of antioxidants to scavenge ABTS^•+^) ranged between 0.18 ± 0.01 and 25.28 ± 1.07 *µ*mol Trolox/g DW for the water-soluble fractions, with black rice, organic black rice, buckwheat, and red glutinous rice exhibiting the highest values. For fat-soluble fractions, the TEAC values varied from 0.06 ± 0.04 to 5.22 ± 0.29 *µ*mol Trolox/g. The antioxidant capacities of cereals showed a significant correlation between the FRAP value and the TEAC values. A strong correlation between antioxidant capacity and total phenolic content (Folin-Ciocalteu) was also obtained, indicating that phenolic compounds mainly contribute to the antioxidant capacities of these cereals [105].

The total antioxidant capacity and phenolic content were assessed for 70 medicinal plant infusions.* Melissae folium* infusions exhibited a ferric reducing antioxidant power greater than 20 mmole Fe(II)/L and a phenol antioxidant coefficient greater than 3. The DPPH radical scavenging ability of* Melissae folium* phenolics was close to that of catechin. With respect to ABTS radical cation scavenging,* Melissae folium* phenolics exhibited superior efficacy in comparison to Trolox and vitamin C [212].

Species belonging to Malvaceae family (*Sidastrum micranthum* (A. St.-Hil.) Fryxell,* Wissadula periplocifolia* (L.) C. Presl,* Sida rhombifolia* (L.) E. H. L., and* Herissantia crispa* L. (Brizicky)) were investigated for the total phenolic content, DPPH radical scavenging activity, and Trolox equivalent antioxidant capacity. The antioxidant activity of the crude extract, aqueous and organic phases and isolated flavonoids, kaempferol 3,7-di-O-*α*-L-rhamnopyranoside (lespedin), and kaempferol 3-O-*β*-D-(6′′-E-p-coumaroyl) glucopyranoside (tiliroside) was assessed. A firm correlation was noticed between total polyphenol content and antioxidant activity of the crude extract of* Sidastrum micranthum* and* Wissadula periplocifolia*; this was not the case for* Sida rhombifolia* and* Herissantia crispa*. The ethyl acetate phase exhibited the best total phenolics content, as well as antioxidant capacity in DPPH and TEAC assays, followed by the chloroform phase. To lespedin, present in the ethyl acetate phase of* W. periplocifolia* and* H. crispa*, no significant antioxidant activity has been ascribed (IC_50_: DPPH: 1,019.92 ± 68.99 mg/mL; TEAC: 52.70 ± 0.47 mg/mL); tiliroside, isolated from* W. periplocifolia*,* H. crispa*, and* S. micranthum*, had small IC_50_ (1.63 ± 0.86 mg/mL), proving better antioxidant capacity as provided by TEAC method [213].

The antioxidant properties of* Diospyros bipindensis* (Gürke, used in Baka traditional medicine against respiratory diseases) stembark were assessed by ABTS, DPPH, and ORAC assays. The antioxidant properties that contribute to the bioactivity of the plant extract were mainly imparted by ismailin [179].

During the investigation of the antioxidant potentials of some cereals and pseudocereals, polyphenol dry matter extracts from seeds of buckwheat, rice, soybean, amaranth, and quinoa (obtained with 1.2 M HCl in 50% methanol/water) showed better inhibition of lipid peroxidation than the ones extracted with 50% methanol/water and were close to the antioxidant activity of BHT at concentration of 0.2 mg/mL. The antioxidant activities of seed extracts determined by DPPH^•^, ABTS^•+^ scavenging, and *β*-carotene bleaching proved strong correlation with the total polyphenols assessed by Folin-Ciocalteu assay. It has been concluded that proteins do not significantly contribute to the samples' antioxidant activity and that buckwheat followed by quinoa and amaranth are the most proper as cereal substitutes [214].

A series of foods usually consumed in Italy were analysed for their antioxidant capacity, by TEAC, TRAP (relying on the protective action of antioxidants, over the fluorescence diminution of R-phycoerythrin in a monitored peroxidation reaction), and FRAP. Among vegetables, spinach had the highest antioxidant capacity in the TEAC and FRAP assays, followed by peppers, while asparagus had the greatest antioxidant capacity in the TRAP assay. Among fruits, berries (blackberry, redcurrant, and raspberry) possessed the highest antioxidant capacity in all assays. With respect to beverages, coffee had the greatest total antioxidant activity regardless of the technique, followed by citrus juices. As for oils, soybean oil had the highest antioxidant capacity, followed by extra virgin olive oil, whereas peanut oil proved less effective [215].

The* in vitro* antioxidant activity of wines has been investigated by a series of determinations such as ORAC, ABTS, DPPH, and DMPD quenching. Also, the total phenolic index was assessed for the 41 samples subject to analysis. Red wines necessitated solid phase extraction to discriminate three main fractions. ABTS, DPPH, and ORAC provided the same reactivity ranking of the analysed fractions: fraction 2 (flavan-3-ol and anthocyanins) showed the best activity followed by fractions 1 (phenolic acids) and 3 (flavonols). Nevertheless, a much reduced reactivity of fraction 2 components (anthocyanins and flavanols) towards DMPD^*∙*+^ was noticed which is consistent with the lower correlation with the total phenolic index and the smaller difference (red versus white wines) in comparison to the other methods' results [216].

#### 4.2.2. Studies Relying on Both Enzyme-Based and Nonenzyme Assays

The antioxidant activity of the ethyl acetate and* n*-butanol extracts, along with that of seven flavonol glycosides isolated from* Dorycnium hirsutum* aerial parts, was assessed using the DPPH method and the lipoxygenase assay. With respect to the inhibition activity towards the DPPH radical, kaempferol 3-O-*α*-L-rhamnopyranoside possessed the highest antioxidant activity (1.226 Trolox equivalents). The lipoxygenase assay has been also performed, in the presence of linoleic acid and commercial lipoxygenase at pH = 6.80, and the hydroperoxide generation was monitored at 235 nm. It was proven that kaempferol 3-(4′′-O-acetyl)-O-*α*-L-rhamnopyranoside-7-O-*α*-L-rhamnopyranoside exhibited the best antioxidant protection activity in the lipid peroxidation system. The butanolic extract proved less active than the ethyl acetate residue, which confirmed the higher flavonoid level assessed in the latter [217].

The scavenging properties of the DPPH radical and the xanthine oxidase inhibition activity were determined for methanol extracts of* Lantana camara* obtained from different plant parts. The absorbance diminution of the DPPH solution was measured at 517 nm, and the XO inhibition assay was based on uric acid production estimated according to the absorbance increase at 290 nm. Allopurinol (XO inhibitor) was used as positive control. The DPPH assay showed that the leaf extract had the best antioxidant activity, and the highest phenolic content was also found in the leaves: 245.50 ± 3.54 mg gallic acid/g [180].

The* in vitro* antioxidant capacities of methanolic extracts (80%) prepared from* Cornus mas *L.,* Diospyros kaki* L., and* Laurocerasus officinalis *Roem were tested by a series of recognized methods: DPPH, superoxide radical scavenging, FRAP, CUPRAC, metal-chelating capacity, *β*-carotene bleaching test in a linoleic acid emulsion system, and TEAC [186]. For the superoxide radical scavenging, the extract was subject to reaction with a substrate solution containing sodium xanthine and 2-(4-iodophenyl)-3-(4-nitrophenol)-5-phenyltetrazolium chloride. Xanthine oxidase was used as biocatalyst and the absorbance increase was monitored at 505 nm. Gallic acid was used as reference phenolic [143, 186]. The Folin-Ciocalteu assay of total phenolic content involves sample reaction with a mixture of phosphomolybdate and phosphotungstate in the presence of sodium carbonate 20%. Absorbance readings of the blue molybdenum-tungsten complex formed in the presence of reducing phenolics are taken at 765 nm after incubation, with gallic acid as [218].* Diospyros kaki* yielded the best results, except for the *β*-carotene bleaching assay. Also, good correlation was obtained between the phenolic profile and antioxidant activity. No metal-chelating activity was shown [186].

The isolation and characterization of bioactive polyphenolic compounds from bitter cumin (condiment and also stimulant, carminative, and astringent in traditional Ayurvedic medicine) were performed, by various spectrophotometric determinations, to assess their scavenging activities. The bitter cumin seed extract showed enhanced antioxidant activity at *µ*g amounts, trapping effectively DPPH^•^, lipid peroxyl, hydroxyl, and superoxide anion radicals. Superoxide anions were generated in the samples containing nitroblue tetrazolium, nicotinamide adenine dinucleotide-reduced, and phenazine methosulphate. The absorbance taken at 560 nm decreased in the presence of cumin extracts, pointing towards superoxide anion scavenging activity. The measurement of lipid peroxidation activity in rat liver microsomes was performed incubating microsomal protein with ferrous sulphate and ascorbic acid, yielding malonyl dialdehyde, which led to the assessment of TBARS in the presence of thiobarbituric acid with readings taken at 535 nm. The presence of cumin antioxidants led to the decrease of microsomal lipid peroxidation, assessed in the presence of soybean lipoxygenase and linoleic acid. The absorbance due to lipid hydroperoxides was measured at 234 nm. Cumin phenolics decreased lipid peroxidation and proved radical scavenging ability in Fenton OH-initiated DNA damage [106].

Twelve methanolic extracts from rosemary leaves harvested from different locations of Turkey at four different times of the year were analysed for their radical scavenging capacities and antioxidant activities by applying different techniques: DPPH radical scavenging activity, Trolox equivalent antioxidant capacity, and reversing H_2_O_2_-induced erythrocyte membrane lipid peroxidation. Human erythrocyte superoxide dismutase and catalase activities, after* in vitro* incubation with the extracts, were also tested in order to check altered enzymatic efficiency.* Rosmarinus officinalis* samples were collected from three different locations, namely, Canakkale (southern Marmara region, the coolest climate), Izmir (Aegean region, moderately hot), and Mersin (Eastern Mediterranean region, the hottest), on four different intervals as follows: December 2003 (denoted as C-S1, I-S1, and M-S1, resp.), March (C-S2, I-S2, and M-S2), June (C-S3, I-S3, and M-S3), and September 2004 (C-S4, I-S4, and M-S4). TEAC values ranged between 11.7 and 5.3 mmol Trolox/kg FW, with Canakkale–March samples having the highest value (11.7 mmol/kg). With respect to the H_2_O_2_-forced human erythrocyte membrane lipid peroxidation test, most of rosemary extracts acted efficiently except for C-S2, I-S1, IS2, I-S3, and I-S4. I-S2 and I-S3 even exhibited prooxidant activity and caused an increase of MDA. The M series together with C-S4 have been proven to be the most active antioxidants in the* in vitro* test. M-S4 extract showed closest effect to the three reference antioxidants: BHT, vitamin C/vitamin E mixture, and quercetin. All extracts caused significant increases in SOD activity, except C-S1, I-S1, I-S4, and M-S4. M series extracts did not affect CAT activity, while C and I series significantly decreased CAT activity.

All extracts proved high ability to quench the free radicals (DPPH and ABTS) and to inhibit malonyl dialdehyde formation. On the whole, the obtained results revealed that the plants harvested in September possess higher levels of active constituents and superior antioxidant activities in comparison to those harvested in other year seasons. Plants harvested from the Izmir region had lower total phenol and active constituent levels and hence smaller antioxidant activity. These differences were assigned to the various compositions in bioactive compounds characterizing the plants harvested from different locations and in various year seasons: the rosmarinic acid contents in the Izmir and Mersin samples increased in summer, with a maximum in September (30.4 mg g^−1^), and decreased in December, attaining a minimum in March (0.4 mg g^−1^). In the Canakkale samples, the rosmarinic acid level raised progressively from December to September. As in the case of rosmarinic acid, the levels of carnosol peaked in September and then diminished stepwise, attaining a minimum in March. For carnosic acid, seasonal differences were not as significant as for rosmarinic acid and carnosol: all September samples possessed higher levels of carnosic acid. Mersin samples exhibited the highest and lowest carnosic acid levels in September and March, respectively [104].

111 samples of yerba mate from three (Southeast, South Central, and Metropolitan Area of Curitiba) regions of the Brazilian state of Parana were subject to analysis of the total phenolic content (TPC) by near infrared spectroscopy. Multivariate calibration models were developed to assess the TPC from the NIR spectra using partial least squares regression. Namely, the characteristic spectral signal of phenolic groups (4.670 cm^−1^) led to the development of multivariate models for quantifying total phenolics. The reference experimental results and those predicted by the partial least squares regression model obtained for the 26 samples ranged between 27.28 and 44.55 mg g^−1^ [219].

In another study, the antioxidant action of different flavonoids (quercetin, glabridin, red clover extract, and the isoflavones mixture Isoflavin Beta) was investigated to assess the appropriateness as topical formulation against free radicals-induced damage. Horseradish peroxidase catalyses luminol oxidation to 3-aminophthalate by H_2_O_2_, followed by light emission at 428 nm, at pH 7.40, in 0.1 M phosphate buffer. The reduction of chemiluminescent signal in the presence of antioxidants takes account on their antioxidant potential. All samples proved their capacity to inhibit oxidative damage, as depending on quercetin and beta isoflavin level. The highest chemiluminescence inhibition was noticed for glabridin and dry red clover extract [220].

### 4.3. Electrochemical Techniques

#### 4.3.1. Linear Sweep Techniques: Cyclic Voltammetry

The antioxidant capacity of dry extracts of green tea, black tea, rosemary, and coffee, acerola and açaì, was assessed voltammetrically at a glassy carbon electrode. Methanol proved better efficacy in extracting antioxidant principles. The antioxidant capacities given by anodic area on the voltammogram and expressed as mg ascorbic acid equivalents ranked as follows: green tea > black tea > rosemary > arabica coffee > herb tea > acerola > quality tea > açaì [221].

A cyclic voltammetric study of the electrooxidation of phenolic compounds present in wine revealed a pivotal influence of the structure on the propensity to undergo oxidation. Compounds with an orthodiphenol group (catechin, epicatechin, quercetin, and gallic, caffeic, and tannic acids) and morin possess the greatest antioxidant potential and are oxidized at low potentials at about 400 mV. Ferulic acid, trans-resveratrol, and malvin as well as vanillic and p-coumaric acids that have a solitary phenol moiety in many cases close to a methoxy function are less electrooxidizable. Anthocyanins determine the existence of a peak at 650 mV. It was also assessed that phenolics that imparted a first peak in the 370–470 mV range on the voltammogram can be secondly oxidized at around 800 mV: catechin and epicatechin by reason of the meta-diphenol groups on the A-ring, quercetin owing to the -OH group on the C-ring, and gallic acid due to the third -OH group placed next to the orthodiphenol moiety oxidized during the first wave [222].

A voltammetric electronic tongue system composed of an array of modified graphite-epoxy composites and a gold microelectrode was applied in the qualitative and quantitative analysis of polyphenols in wine. Samples were analysed by cyclic voltammetry and did not necessitate sample pretreatment. The analytical responses were processed by discrete wavelet transform in order to compress and extract the essential features of the voltammetric analytical responses. External test subset samples results correlated well with the ones furnished by the Folin-Ciocalteu assay and UV absorbance polyphenol index (I_280_) for amounts from 50 to 2400 mg L^−1^ gallic acid equivalents [223].

BHA, BHT, and TBHQ in edible oil samples were assessed by first derivative voltammetry at glassy carbon electrode modified with gold nanoparticles. First derivative pretreatment was applied to the linear sweep voltammetric signals, aiming at minimizing noise influence. The values of the peak potentials of 0.273, 0.502, and 0.622 V allowed discrimination between TBHQ, BHA, and BHT with detection limits as low as 0.039, 0.080, and 0.079 *μ*g mL^−1^ for the three aforementioned analytes. BHA content ranged between 19.3 *μ*g g^−1^ in rapeseed oil and 56.6 *μ*g g^−1^ in sesame oil. BHT was only found in blend oil at a level of 20.7 *μ*g g^−1^. TBHQ level ranged between 26.8 *μ*g g^−1^ in rapeseed oil and 48.4 *μ*g g^−1^ in corn oil. Interference studies indicated that the determination of all three compounds in commercial edible oil samples was not significantly affected by the common interferents, 2-fold ascorbic acid and 10-fold vitamin E; phthalate and citric acid concentrations exerted no significant influence on the peak currents of the three synthetic food antioxidants: ascorbic acid peak appeared at 0.437 V and did not hinder the analytical signals of the three analytes. Vitamin E and phthalate and citric acid did not show peaks in the potential range of 0.10–0.80 V. Metal ions (K^+^, Na^+^, Ca^2+^, Fe^2+^, Mg^2+^, and Zn^2+^) and some of the most significant anions (Cl^−^, I^−^, SO_3_^2−^, SO_4_^2−^, NO_3_^−^, CO_3_^2−^, PO_4_^3−^, and CH_3_COO^−^) did not interfere also up to a 100-fold increase in their concentrations [112].

#### 4.3.2. Differential Pulse Voltammetry

Sesamol and lignans contents in sesame, tahina, and halva were assessed by polarography and stripping voltammetry. Differential pulse polarography used a capillary hanging mercury drop electrode. In cathodic stripping, sesamol reacts forming a reduced derivative, which is oxidized, yielding a cyclic voltammetric peak. The cathodic stripping voltammetric assessment involved preconcentration (when sesamol accumulates to the electrode-surface at −1650 mV) and scanning (when analyte stripping takes place in the potential range of −1650 mV to −2250 mV). Using these electrochemical methods, sesamol was assessed at levels of 0.26–0.32 mg/100 g oil in three varieties of sesame, 10.98–12.33 mg/100 g oil in tahina, and 4.97–9.12 mg/100 g oil in halva [224].

The antioxidant activity of flavonoids (catechin, quercetin, dihydroquercetin, and rutin) was voltammetrically assessed at a glassy carbon working electrode, and the measured redox potentials were correlated to the antioxidant activity results. Differential pulse measurements relied on molecular oxygen cathodic reduction, at 50 mV s^−1^, in the 0–800 mV potential range and for an optimal pulse amplitude of 10 mV. The antioxidant activity coefficient was assessed considering O_2_ reduction current in the presence of added antioxidants and the limiting O_2_ current in the absence of antioxidants. To check reversibility and electrooxidation mechanism, cyclic voltammetric assay was performed for the tested flavonoids (1–10 *μ*mol L^−1^ in phosphate buffer 0.025 M, pH = 6.86) in the potential range of 0–1000 mV, at 50 mV s^−1^. All flavonoids presented reversible peaks in the range of 300 mV–400 mV. The intensity of the CV oxidation peak (assigned to the deprotonation of the catechol moiety, namely, of the 3′-OH electron-donating group) increases with the dihydroquercetin concentration. The peak intensity also depended linearly on the square root of the scan rate, so it was concluded that the oxidation of this flavonoid was limited by mass transfer. Both the easiness of electron transfer reflected by the redox potentials (in CV) and the antioxidant activity (assessed by DPV) correlated with the following trend of increase: rutin–dihydroquercetin–catechin–quercetin [225].

A DNA-modified carbon paste voltammetric biosensor functioned on the basis of DNA layer oxidative insults, induced by Fenton OH^•^ radicals. The electrooxidation of the left unimpaired adenine bases can give an oxidation product able to catalyse NADH oxidation [226, 227]. Antioxidants scavenged hydroxyl radicals and the current which emerged from NADH oxidation increased as a result of a larger number of unoxidized adenine molecules [226]. Ascorbic acid has been proven to be the most effective antioxidant, as it determined the most significant electrocatalytic current increase. Among the analysed beverages, the greatest antioxidant capacity value was exhibited by lemon flavour, namely, 480 ± 20 *μ*M, calculated as per reference to ascorbic acid standard [226].

**Figure 3:**
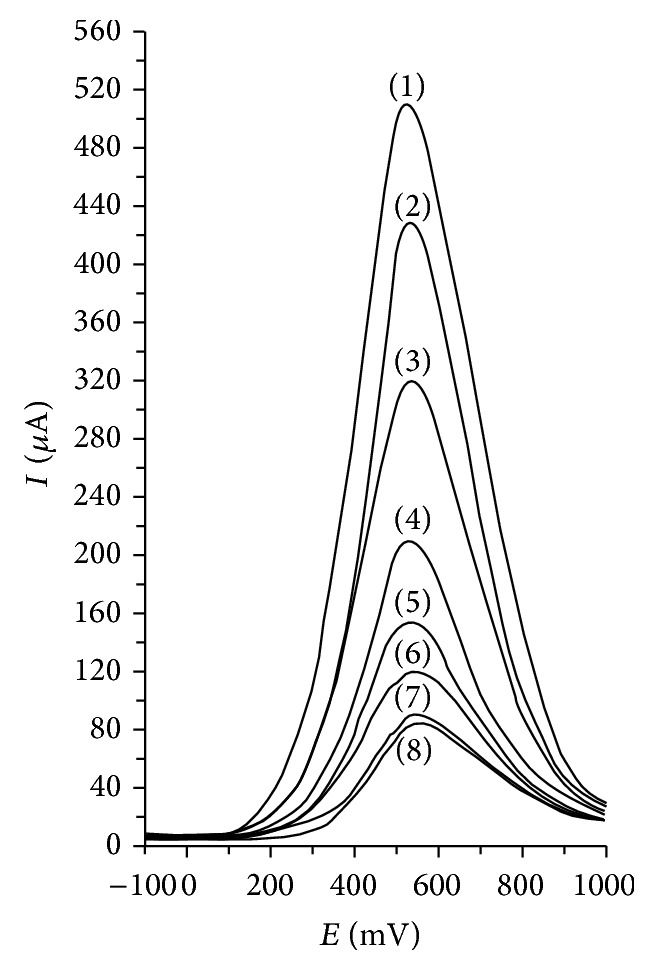
Differential pulse voltammograms at a Pt working electrode for different ascorbic acid concentrations (mM): 20 (1), 15 (2), 10 (3), 5 (4), 2.5 (5), 1.25 (6), 0.625 (7), and 0.31 (8); pulse amplitude, 75 mV, pulse period, 125 ms, and potential scan rate, 50 mV/s [89].

Differential pulse voltammetry a Pt working electrode allowed for ascorbic acid quantitation in fruit juices, based on its electrooxidation, as presented in Figure 3 [89].

#### 4.3.3. Square Wave Voltammetry

Square wave voltammetry at disposable screen-printed carbon electrodes was developed for the polyphenol antioxidant screening in freshly squeezed blackcurrant and strawberry juices from various cultivars and at different maturity stages. Prior to the electrochemical assessment, the anthocyanins and nonanthocyanins were separated by solid phase extraction. It was proven that the charges passing to 500 and 1000 mV correlated well with the antioxidant activity, as well as with anthocyanin and ascorbate levels. The use of disposable screen-printed sensors was able to surpass the shortcoming of electrode deactivation due to fouling by the polymeric film formed through coupling of phenoxyl radicals during electrooxidation. Nevertheless, it was asserted that phenolics other than anthocyanins, with low formal oxidation potentials, should be quantified on the basis of sensor cumulative responses at 300 mV. Blackcurrant juices possessed high oxidation peaks at low potentials (<400 mV) and so enhanced antioxidant capacities [228].

The phenolic content of extra virgin olive oils was assessed by an array of 12 voltammetric electrodes: five lanthanide bis-phthalocyanines-based sensors, six polypyrrole-based sensors, and one unmodified carbon paste electrode. Apart from the peaks related to phthalocyanine, a peak due to the redox process associated with the polyphenolic fraction was also present as a shoulder in the domain of 300–500 mV (assigned as first peak). The oxidation of lanthanide bis-phthalocyanines complexes occurs at 0.55 V in KCl electrolyte and at 0.66 V in the oil extract, so the antioxidant features of polyphenols render the oxidation of phthalocyanine more difficult. The electrocatalytic efficacy of phthalocyanines enables a more facile oxidation of phenols: for the extract with the highest polyphenol content, when using a bare carbon paste electrode, the peak assigned to polyphenols appeared at 0.68 V and at 0.5 V when using a praseodymium bis-phthalocyanine electrode. The polyphenol content of the extracts ranged between 403.06 mg kg^−1^ and 990.25 mg kg^−1^ [209].

Square wave voltammetry and cyclic voltammetry at different pH values was performed to investigate the antioxidant capacity of cashew nut shell liquid components, such as cardol, cardanol, and tert-butylcardanol which were characterized by lower oxidation potentials in comparison to BHT (Epa = 0.989 V). Cardol possessed the smallest Epa (0.665 V) and hence best antioxidant potential among the tested compounds, followed by tert-butylcardanol (Epa = 0.682 V) and cardanol (Epa = 0.989 V). A linear shift of the peak potential to more negative values with the pH increase was noted for all antioxidant compounds. Increasing the pH of the electrolyte resulted in a nonlinear decrease of the peak currents, so the highest values were obtained at pH = 2.0 [229].

#### 4.3.4. Stripping Voltammetry

Adsorptive stripping voltammetry has been applied for the determination of caffeic acid at a Pb film electrode. The working electrode was obtained in situ on a glassy carbon basis. The analyte accumulates by adsorption on the lead film electrode and is subsequently electrooxidized during the stripping step. The analytical signal depended linearly on caffeic acid concentration in the range of 1 × 10^−8^ to 5 ×10^−7^ M with a detection limit of 4 × 10^−9^ M in acetate buffer pH = 4.0. By operating in the square wave voltammetric mode, this technique proved viable in the determination of caffeic acid in the herbs of* Plantago lanceolata*. The caffeic acid amount obtained by the developed voltammetric method was 107.4 *µ*g/g of dried plant with RSD of 2.95% [93].

#### 4.3.5. Hydrodynamic Techniques: Amperometry

The determination of flavonoids and ascorbic acid in grapefruit (*Citrus paradisi*, antioxidant, antiallergic, and anticarcinogenic) peel and juice has been performed by capillary electrophoresis with electrochemical detection. Hydrodynamic voltammetric measurements aimed at optimizing operational parameters. An applied potential of +0.95 V (versus SCE) was chosen for an optimum signal to noise ratio and a pH of the running buffer of 9.0, because at this value a rapid and efficient separation was obtained between the five flavonoids. 60 mM was the best buffer concentration, with higher values negatively influencing the detection limit. Hesperidin, naringin, hesperidin, naringenin, rutin, and ascorbic acid were separated and quantified in grapefruit juice by capillary electrophoresis with electrochemical detection, comparing the migration times with those of the standards. Hesperidin, naringin, and ascorbic acid were assessed in grapefruit peel extract, with good peak repeatabilities and recoveries [230].

A sequential injection method with amperometric detection was developed for the total antioxidant capacity assessment in commercial instant ginger infusion beverages. The method relied on the decrease of the cathodic current of ABTS^•+^ at a glassy carbon electrode in phosphate buffer, pH = 7.0, after reaction with antioxidants in the sample. The total antioxidant capacity ranged between 0.326 ± 0.025 and 1.201 ± 0.023 mg gallic acid equivalents/g sample [231].

A laccase-based amperometric biosensor allowed for the optimized determination of phenolic content in tea infusions. The enzyme from* Trametes versicolor* was immobilized by entrapment within polyvinyl alcohol photopolymer fixed onto disposable graphite screen-printed electrodes. An oxidation peak was noticed at 270 mV only in the presence of hydroquinone, the enzyme substrate. The amperometric responses of the biosensor were registered for three tested diphenols under the optimal experimental conditions, 0.1 M acetate buffer at pH 4.70, 30°C, and −300 mV. The highest sensitivity was obtained for catechol 18.82 ± 0.76 nA *µ*M^−1^, whereas the lowest one was obtained for resorcinol 0.110 ± 0.002 nA *µ*M^−1^. The equivalent phenol content was comprised between 4.0 and 109.2 mg caffeic acid equivalents/L sample for orange leaves and palo azul infusion, respectively [232].

A laccase-based biosensor aimed at polyphenols determination from* in vitro Salvia* cultures. The biosensor was developed by drop casting 3 mL of laccase solution and stabilization with 0.1% Nafion solution on a screen-printed carbon electrode. Chronoamperometric measurements were performed in 0.1 M phosphate buffer, pH 4.50, at −30 mV versus an Ag/AgCl reference. The results, as rosmarinic acid equivalent content (chosen as standard, as it has been identified as the major phenolic in the samples analysed, by chromatography and MS screening), ranged from 97.8 ± 8.2 *µ*g/g fresh material for* Salvia maxima* to 162.2 ± 11.3 *µ*g/g fresh material for* Salvia verde*, with a limit of detection of 4.2 × 10^−7^ M. The biosensor retains more than 85% of its initial analytical response up to 90 days. The Michaelis-Menten kinetic apparent constant of 8.3 × 10^−6^ M revealed the enzyme affinity for the substrate [110].

A tyrosinase-based biosensor was prepared by enzyme immobilization on single wall carbon nanotubes screen-printed electrodes modified with iron(II) phthalocyanine. The electrochemical behavior of the biosensor was studied with optimization of the parameters: the cyclic voltammograms in catechin solution do not present peaks related to phthalocyanine but exhibit only the peak related to the reduction of the o-quinone that resulted from tyrosinase-catalysed catechin oxidation. This peak points to the retaining of the catalytic activity of tyrosinase after immobilization. The maximum analytical response was obtained at 0.15 V, pH = 7.0, in phosphate buffer solution. The amperometric signal at increasing catechin concentrations was registered, with a sensitivity of 0.937 *µ*A *µ*M^−1^ and a 0.89 *µ*M detection limit. The polyphenol content of green tea samples, as mg catechin, ranged from 19 ± 1.45 to 98 ± 3.86 [233].

A superoxide dismutase-based biosensor allowed for the amperometric antioxidant activity determination in aromatic herbs, olives, and fruits. Superoxide dismutase was immobilized between a cellulose acetate membrane and a dialysis membrane. Enzymic xanthine oxidation yields superoxide radical anion and the disproportionation of the latter by superoxide dismutase results in molecular oxygen and hydrogen peroxide occurrence [234]:

(6) xanthine+H2O+O2⟶uric  acid+2H++O2∙−2O2∙−+2H+⟶H2O2+O2

Finally, the electrochemical response correlatable to the superoxide radical concentration is imparted by H_2_O_2_ oxidation at the Pt anode and 650 mV. As the electroactive superoxide anion radicals are trapped by antioxidants in the sample, the amperometric signal diminishes. The highest antioxidant capacity was exhibited by sage as herb and by medlar, among analysed fruits [234].

#### 4.3.6. Biamperometry

This method relies on recording at a small potential difference, the current intensity between two identical working electrodes, found in a solution where a reversible redox couple (Fe^3+^/Fe^2+^, I_2_/I^−^, Fe(CN)_6_^3−^/Fe(CN)_6_^4−^) is present. The analyte reacts with the indicating redox couple: the selectivity of the technique depending on the specificity of the reaction involving the oxidized form of the redox pair and the antioxidant [158].

Particularly in DPPH^•^/DPPH biamperometry, antioxidants react with DPPH^•^ (radical form) decreasing its concentration and generating DPPH (reduced form). The DPPH^•^ reduction at one electrode gives rise to a cathodic current proportional with the concentration of the radical form, whereas the oxidation of DPPH at the other electrode yields an anodic current proportional with the molecular form concentration. When employed working conditions are as such, for the radical form concentration to be smaller than the one proper to the molecular form, cathodic current is limited by the lower concentration of DPPH^•^ radical in the indicating mixture. DPPH^•^/DPPH biamperometry was employed in the analysis of tea, wine, and coffee using glassy carbon electrodes [158]. ABTS^•+^/ABTS biamperometry enables analysis of juices, tea, and wine [159], as well as wines and spirits with excellent sensitivity, 0.165 nA/*μ*M Trolox [160].

Samples of Brazilian woods and oak (*Quercus* sp.) extracts were subject to ceric reducing antioxidant capacity (CRAC) analysis at a boron-doped diamond film electrode, relying on Ce^4+^/Ce^3+^ redox couple. Chronoamperometric determinations enabled quantification of the decrease of Ce^4+^ concentration, which was caused by its reduction by antioxidants present in the sample. The following variation of the antioxidant activity of analysed extracts was observed: oak (*Quercus* sp.) (1.73) > cabreuva-vermelha (*Myroxylon balsamum*) (1.05) > cabreuva (*Myrocarpus frondosus*) (0.90) > imbuia (*Octea porosa*) (0.71) > pequi (*Caryocar brasiliense*) (0.31) [235].

The ceric reducing antioxidant capacity assay was also exploited given its direct electron transfer facility for determining the antioxidant capacity of eight antioxidant compounds. The developed technique was based on observing the decrease of Ce^4+^ concentration after its reaction with antioxidants. The following trend of variation of antioxidant capacities resulted from this comparative investigation, which relied on chronoamperometric measurements: tannic acid > quercetin > rutin > gallic acid ≈ catechin > ascorbic acid > butylated hydroxyanisole > Trolox. The results were consistent with those furnished by the applied conventional FRAP assay [236].

Vitamin C assessment relied on the oxidation of the analyte at acidic pH by I_2_/I^−^ employed as oxidizing agent. The biamperometric detection of the amount of iodine consumed allowed for the assessment of vitamin C with a linear range of analytical response comprised between 5 × 10^−5^ and 5 × 10^−4^ M: a 1.08% RSD (*n* = 10; *c* = 2.5 × 10^−4^ M) and high throughput of 60 samples h^−1^ [237].

#### 4.3.7. Potentiometric Assay

The analytical signal represented by the potential change is the result of the variation of an ionic species concentration. A flow injection potentiometric method was developed to rapidly and reproducibly evaluate the antioxidative ability characteristic for several aqueous plant extracts. This potentiometric technique relies on recording the potential shift in the ferricyanide/ferrocyanide mediator system in the presence of antioxidants, which react with the oxidized form of the redox pair, modifying the concentration ratio between the oxidized and reduced forms. The developed potentiometric method used as detector a Pt electrode transducer based on logarithmic dependence, which provided the antioxidant activity of several hydrosoluble antioxidants (ascorbic acid, pyrocatechol, pyrogallol, caffeic acid, chlorogenic acid, gallic acid, tannic acid, uric acid, l-cysteine, and Trolox). The total antioxidant activity of aqueous fruit extracts was comprised between 0.066 ± 0.002 ascorbic acid equivalents (for lemon) and 0.490 ± 0.001 (for orange). The tea infusions' values ranged between 3.60 ± 0.1 ascorbic acid equivalents for Dolche vita and 18.0 ± 0.2 for Sweet osman [161].

A linear potentiometric response versus ascorbic acid at an iodine-modified platinum electrode was obtained between 1.0 × 10^−5^ and 1.0 × 10^−3^ M in model solutions. The study also assessed the contribution of ascorbic acid to the total antioxidant capacity of aqueous extracts of hips, hop cones, and lemon juice, namely, 26.0, 0.16, and 15%, respectively [238].

In Table 3, a synoptic view of relevant examples of total antioxidant capacity assessment in plants is given.

## 5. Critical Perspective and Conclusive Aspects Regarding Performances of Extraction and Detection Mode

As previously discussed in this review, the first step is represented by the choice of the adequate extraction method and solvent, and here the nature of the sample and contained active principles, as well as the analytical technology subsequently applied, should be considered. Detailed discussions devoted to the choice of the proper solvent as well as to the extraction methodology are given in specific cases.

Ethanol, methanol, and water, due to their polarity, favor the extraction of polar substances such as phenolics and flavonoids. Owing to its low toxicity and being an organic solvent, ethanol is employed with high frequency. Methanol toxicity hinders somehow its use. Nonpolar solvents like ether, as well as solvents endowed with low polarity (chloroform, ester, acetone, etc.), are proper for particular cases and have limited availability [18]. Phenolic compounds from* M. pubescens* were extracted by maceration employing comparatively solvents with various polarities. It has been revealed that the use of aqueous methanol (50%), aqueous ethanol (50%), and aqueous acetone (50%) imparted the highest antioxidant capacity values. The aqueous methanol (50%) and the aqueous ethanol (50%) extracts possessed the highest polyphenol content [239]. The differences between two techniques applied to phenolic extraction from* Ouratea lucens* and* Acomastylis rossii*, namely, conventional sonication (shaker) bath and homogenizer method, revealed a better performance of the latter. Sonication efficacy depends on the cell wall disruption during grinding, and the better homogenizer performances were attributed to efficacious cell wall breakdown [240].

Three genotypes of horseradish roots were subject to extraction employing conventional solvent, as well as Soxhlet extraction. The solvents endowed with the most potent extractive power were ethanol and ethanol/water mixture. The total phenolic content obtained in Soxhlet extracts proved superior to the one obtained by conventional solvent extraction. Nevertheless, the DPPH^•^ scavenging potential was not increased. So, in this case, by applying Soxhlet extraction, compounds other than antioxidants can take part in the extraction [241]. Soxhlet extraction performances were compared to those of maceration in the case of cumin seeds, and it was concluded that the greatest polyphenols and flavonoid contents were obtained following Soxhlet extraction, whereas employing maceration resulted in better antiradical activity [113].

Vortex extraction led to superior performances when compared to sonication and shaking, for phenolics extraction from oregano leaves, as proved by the results of total phenolic content assay and scavenging activity determination by DPPH^•^. Its efficacy has been proven to be solvent dependent, with best performances being obtained with acetone : water [242].

The comparative analysis of both conventional and nonconventional extraction techniques applied on the same plant material (*Quercus infectoria* extract) revealed that the supercritical CO_2_ extraction resulted in lower extraction yield in comparison to Soxhlet conventional technique, though the antioxidant capacity and selectivity with respect to total phenolics given by supercritical CO_2_ extraction has been proven to be higher than the one imparted by Soxhlet extraction [243].

Various extraction methods (refluxing, sonication bath, ultrasonic homogenizer, and microwave) were applied to the aerial roots of* Rhaphidophora aurea*. The ultrasonic and microwave assisted extraction gave maximum efficiency reduced the costs and the time, limited solvent use, and resulted in a good yield compared to the other investigated techniques. It has been concluded from this comparative investigation that the ultrasonic homogenizer extraction method can be regarded as standard technique for ethyl acetate and ethanol extraction, whereas microwave assisted extraction has been proven to be the most appropriate when water is used as solvent [244].

Grape resveratrol extraction performances were investigated by liquid-liquid extraction, solid-liquid extraction, pressurized liquid extraction-solid phase extraction, and supercritical carbon dioxide extraction and in all situations the yield can be improved by postharvest ultrasonication, fungal pathogens,* in vitro* AlCl_3_ treatment, and UV-C radiation [245].

The health benefits of plant-derived products have been already stated: the lipid oxidation delaying ability led to preventing the occurrence of mutagenic and carcinogenic lipid peroxides and aldehydes. Moreover, spices and herbs have been used for years for flavour, aroma, and color preserving [246]. Dried fruits constitute a rich source of antioxidant phytochemicals (namely, phenolics and carotenoids) and have been recently incorporated in fruit-based functional foods. Nevertheless, elaborated studies are still required for the validation of dried fruits benefits [247]. Investigating improved ways for isolation of active phytocompounds, and employing chemometrics in establishing the effective combinations of spices or herb-sourced antioxidants is an increasing trend, in view of the steady high quality requirements that led to constant optimization of analytical techniques [246].

With respect to the analytical methodology, the development of experimental conditions of the working protocols (that should be tuned to the nature of the sample/target compound(s)) and also the interpretation of the results should be carefully considered. A plethora of analytical methods were applied to total antioxidant capacity in plant extracts, yet difficulties may arise when it comes to choosing the most adequate method. It was stressed out that conditions, nature of substrate, and concentration of analysed antioxidants should be as close as possible to those encountered in food or biological media [248]. So, the term antioxidant activity is tightly related to the context of particular reaction conditions [44].

In the case of the radical scavenging antioxidants, the antioxidant-radical reactivity (both rate constant and stoichiometry), antioxidant localization, mobility in reaction media, stability of the antioxidant-derived radical, interrelation with other antioxidants, and metabolism should be measured to thoroughly understand their dynamic action as antioxidants. For instance, vitamin E has been proven to be an efficacious radical scavenger, but it was asserted that it possesses weak activity against lipid peroxidation by lipoxygenase. Carotenoids are not efficient in radical trapping but they inhibit single oxygen-induced oxidation [249]. In the presence of transition metal ions, phenolic radical scavengers can even induce oxidative damage to lipids, acting as prooxidants through the aryloxy radical. The latter is formed during the reaction of the phenolics with Cu(II) and can attack the lipid substrate [44], following a radicalic mechanism similar to the one described in Section 1.

The choice of the appropriate method should be grounded on the biomolecules targeted during the oxidation process (lipids, proteins, and nucleic acids). A viable antioxidant protocol necessitates the quantification of more than one property relevant to either foods or biological systems. Antioxidant standardization is required to diminish the discrepancies that may result from only one technique applied to antioxidant assessment [250].

A comparative discussion of the detection mechanism should consider the following aspects. Gas chromatography allows separation and determination of a precise amount of certain antioxidants in different media. HPLC as well as gas chromatography coupled to various detectors also results in detecting individual, particular antioxidant compounds (ascorbic acid, tocopherols, flavonoids, phenolic acids, etc.). These methods require qualification; they are laborious but benefit from most accurate and efficacious separation and quantitation [13, 251]. HPLC can provide limits of detection and quantification of 0.4 and 1.2 ng/mL such as for the case of linalool, 0.6 and 1.4 ng/mL, and geranyl acetate, respectively [96].

With respect to the methods relying on optical detection, the antioxidant activity is exerted by various mechanisms, so the results furnished by a technique that relies solely on one mechanism may not take account on the actual antioxidant activity value. There are assays referring to various oxygenated/nitrogenated species, methods appliable to both lipophilic and hydrophilic antioxidants, and techniques relying on either hydrogen transfer or single electron transfer [248]. It has been assessed that the ones involving peroxyl radical trapping are the most extensively applied (TRAP, ORAC, beta-carotene bleaching, and chemiluminescence) as this radical species is prevalent in biological media. Nevertheless, these methods relying on hydrogen atom transfer should be corroborated with assays relying on single electron transfer (such as ABTS quenching). On the other hand, single electron transfer-based methods rely on slow reactions that are sensitive to ascorbic acid, uric acid, and polyphenols. Secondary reactions are likely to occur, which may lead to interferences [85, 252]. Among the spectrometric* in vitro* methods, their frequency of application decreases in the order: DPPH scavenging > hydroxyl radical scavenging > superoxide dismutase activity > beta-carotene linoleate bleaching. Recently, reliable results have been obtained by combining* in vitro* method (DPPH assay) with high performance liquid chromatography. Such a DPPH-HPLC online assay especially for natural-sourced antioxidants requires minimum sample preparation [253]. Moreover, it allows hampering of the drawbacks of the simple DPPH technique that uses offline colorimetric detection and for which the small changes in absorbance cannot be quantified [254]. With respect to the* in vivo* techniques, the lipid peroxidase assay is the most frequently used, followed by catalase and glutathione peroxidase [18, 251]. The* in vivo* antioxidant capacity assay uses biological media [255], so the antioxidants usually undergo absorption, transport, distribution, and retention in the biological fluids, cells, and tissues. As during these steps many alterations can occur (e.g., biotransformation during enzymatic conjugation), the experimental protocol has to be cautiously carried out [256, 257]. The discussion* in vivo* versus* in vitro* is of vital importance in these types of assays, as the antioxidant capacity of plants and phytocompounds is influenced by various parameters* in vivo*, such as gut absorption, metabolism, bioavailability, and the presence of other antioxidants or transition metal ions [50], and screening by* in vitro* assays should be complemented by* in vivo* efficacy testing [258]. Moreover, it has been stressed out that once the efficacy of a phytocompound is proven* in vivo*, the mechanisms of action should be subject to analysis* in vitro* to avoid discrepancies that may occur when validating* in vitro* confirmed methods* in vivo* [50].

When it comes to the chemiluminescent assay, the major shortcoming is represented by light emission from other sources [13, 251]. The ORAC technique with fluorescent detection relies on a mechanism regarded as biologically pertinent, as it takes account on the contribution of both lipophilic and hydrophilic antioxidants [85].

Nevertheless, in ORAC assay, only the antioxidant activity against peroxyl radicals is measured, disregarding the other reactive oxygenated species. It has been also asserted that antioxidant molecules present in foodstuffs exert numerous functions, some not related to the capacity to trap free radicals. Also, it has been considered that the values of antioxidant capacity do not give account on all the effects of particular bioactive principles [259]. The values of antioxidant capacity furnished by* in vitro* studies cannot be extrapolated to* in vivo* effects, and the clinical trials testing benefits of dietary antioxidants may result in mixed results. Moreover, it was mentioned that ORAC values expressed as Trolox equivalents give account on both the inhibition time and the extent of oxidation inhibition. Novel versions of the ORAC assay have been developed, which use other substrates as reference (e.g., gallic acid), which makes data comparison not an easy task. These considerations led to ORAC withdrawal from the online catalog of United States Department of Agriculture [259]. So, if ORAC assay is applied as a complementary assay instrument in connection with other analytical techniques, data comparison has to be performed using the same standard, clearly defined units of expressing the results, distinguishing between dried and fresh foods and juices or other processed foods. Measuring* in vitro* antioxidant properties remains useful, as far as the benefits are* also* related to what happens outside human body.

The type of the prevalent antioxidant class present or the reference antioxidant chosen plays important roles with respect to the correlation of results obtained by different spectrometric methods: in the case of guava fruit methanol extract, the ABTS, DPPH, FRAP, and ORAC methods gave close results. The antioxidant activity of methanol extract exhibited good correlation with ascorbic acid or total phenolics, with best correlation being shown by FRAP results with both ascorbic acid and total phenolic content. On the other hand, ABTS, DPPH, and FRAP results for methanolic extracts were negatively correlated with total carotenoids. The antioxidant capacity of dichloromethane extract (that took account on lipophilic antioxidants) was low, compared to antioxidant activity of methanol extract. So, hydrophilic ascorbic acid and phenolics have been proven to be the main contributors to antioxidant capacity of guava fruit [260]. In Folin-Ciocalteu total phenol assay, it has been assessed that some nonphenolic compounds (e.g., ascorbic acid) that can transfer electrons to phosphomolybdic/phosphotungstic complex in alkaline media may interfere with the results. This shortcoming can be minimized by extraction with 95% methanol applied to plant tissue [261].

Several correlations have been reported between the results obtained at the application of DPPH^•^, ABTS^•+^, and FRAP and total polyphenol content assays as follows: positive correlation between TPC-DPPH^•^ (0.8277), TPC-ABTS^•+^ (0.8835), and TPC-FRAP (0.9153) [262]. Also, correlations have been established between* the antioxidant capacity values reported to different standard antioxidants*: the ABTS^•+^ antioxidant capacities of basil in 57% ethanol extract were 47.27 ± 2.16 mg Trolox equivalents/g DW or 31.17 ± 1.42 mg ascorbic acid equivalents/g DW (white holy basil) and 65.86 ± 5.51 mg Trolox equivalents/g DW or 43.43 ± 3.63 mg ascorbic acid equivalents/g DW (red holy basil). The DPPH^•^ values of 57% ethanol extract of basil were also expressed as per reference to different standards: 5.41 ± 0.04 mg Trolox equivalents/g DW or 4.59 ± 0.03 mg ascorbic equivalents/g DW (white holy basil) and 6.23 ± 0.19 mg Trolox equivalents/g DW or 5.28 ± 0.16 mg ascorbic equivalents/g DW (red holy basil) [263]. The higher values furnished by ABTS^•+^ assay were explained by the fact that compounds which have ABTS^•+^ scavenging activity may not be endowed with DPPH^•^ scavenging potential [264]. Moreover, it was found that some products of ABTS^•+^ scavenging reaction may exert a higher antioxidant capacity than the antioxidants initially present in the reaction medium and can react with ABTS^•+^ [265].

The results of total antioxidant capacity assay of yerba mate* (Ilex paraguariensis*) ethanol extracts evaluated by DPPH^•^ method were expressed as ascorbic acid equivalents or Trolox equivalents (in mass percentage, g% dry matter), trying to facilitate a comparative assessment: 12.8–23.1 g Trolox equivalents % dry matter and 9.1–16.4 g ascorbic acid equivalents % dry matter [266].

Rapidity, lower cost, simpler instrumentation, and oxidation potential value of each particular sample component evaluated with the same accuracy (irrespective of the antioxidant potency, in conditions of efficient peak separation) are reported advantages of electroanalysis versus spectrophotometry. It was asserted that, in DPPH^•^ photometry, the absorbance variations can be subject to more inaccuracy, whereas in voltammetry deviations in the peak potential value smaller than ±3 mV were noticed. Therefore, in photocolorimetry, at low antioxidant capacity values, the results may be prone to a greater uncertainty [267]. Moreover, in such spectrophotometric assays, there can be compounds other than antioxidants that can contribute to the measured analytical signal at the respective wavelength.

Nevertheless, in the case of samples rich in various phenolic classes and possessing different electrooxidation potentials (e.g., wines), *Q*_500_ value as analytical signal does not give account on less oxidizable components, such as phenolics with more elevated oxidation potentials (e.g., 800 mV) [268, 269]. Step voltammetric methods like differential pulse or square wave voltammetry have improved resolution versus linear sweeping methods (cyclic technique), as charging current is minimized [269]. In differential pulse voltammetric polyphenol assessment, the results exhibited a tight correlation with the antioxidant activities furnished by photocolorimetry [154]. Excellent sensitivity is obtained at caffeic acid stripping voltammetric assessment operating in square wave mode: a detection limit of 4 × 10^−9^ mol/L in acetate buffer, pH = 4.0 [93]. Amperometric biosensors have the advantage of enzyme specificity, accuracy, and fastness imparted by electrochemical detection. The drawback of difficultly electrooxidizing high molecular mass antioxidants at fixed potential can be solved by changing the biocatalyst or by the use of mediators that diminish the working potential [269, 270]. Biamperometric techniques provide enhanced selectivity that depends on the specificity of the reaction involving the antioxidant and the oxidized form of the redox couple [158, 271]. The potential value imposed should be strictly controlled, since an increase of the latter could lead to interference of electroactive compounds other than antioxidants that might react at the electrode [272].

Employing minimum two analytical techniques (relying on different mechanisms) and applying three sample dilutions are generally recommended [273, 274]. Hence, a rigorous evaluation of antioxidant capacity should not be restricted to a simple antioxidant test and should consider the variability factors that influence the final value: for instance, the consistency between phenolic content and antioxidant capacity assessed, as well as the techniques' performances, has been proven to be dependent on the nature of the sample, its composition and pH (the latter imparting the existence of protonated or deprotonated form of biocompounds), extraction, and analytical method applied. It was concluded that several test procedures may be necessary to ensure viable antioxidant activity results [3].

Researches focusing on free radicals, plant extracts, plant-derived antioxidants in foodstuffs, and biological media should be accompanied by validation of biological markers meant to define the efficacy of antioxidant compounds in diet [275]. The comparative evaluation of antioxidant potentials reported by different laboratories should consider the significant differences in sample pretreatment, extraction, and final value expression mode [276].

## Figures and Tables

**Table:** (a) Hydrogen atom transfer (HAT)

Corresponding method of assay	Mechanistic description
TRAP (Total Radical Trapping Antioxidant Parameter) assay ORAC (Oxygen Radical Absorbance Capacity) assay Beta carotene/crocin bleaching method Inhibition of induced low-density lipoprotein peroxidation assay Chemiluminescence quenching, due to luminol-derived radicals scavenging by antioxidants	ArOH + X^•^ *⟶* ArO^•^ + XH An antioxidant (e.g., phenolic compound ArOH) directly interacts with a free radical (X^•^), yielding a phenolic radical species derived from the antioxidant molecule ArO^•^, and a neutral species XH. The antioxidant facility to follow HAT mechanism is correlated with low bond-dissociation enthalpy [117]. The presence of dihydroxy functionality imparts good hydrogen donation abilities, correlatable with low bond-dissociation enthalpy values [118].

**Table:** (b) Single electron transfer (SET)

Corresponding method of assay	Mechanistic description
DMPD (N,N-dimethyl-p-phenylenediamine) method FRAP (ferric reducing antioxidant power) assay CUPRAC (cupric reducing antioxidant capacity) method PFRAP (potassium ferricyanide reducing power) method	ArOH + X^•^ *⟶* ArOH^•+^ + X^−^ SET assays rely on the capacity of an antioxidant ArOH to reduce the radical species X^•^ by electron donation, which is accompanied by the color change of the radical solution. Low adiabatic ionization potentials are correlated with good electron transfer abilities [117]. Extended delocalization and electron conjugation result in low ionization potentials [118]. Also, pH increase (deprotonation) favors electron transfer.

**Table:** (c) Mixed HAT and SET

Corresponding method	Mechanistic description
DPPH (2,2-diphenyl-1-picrylhydrazyl) scavenging method	Hydrogen atom transfer and sequential proton-loss electron transfer (SPLET), also designated proton-coupled electron transfer (PCET) [119, 120], were both confirmed as being thermodynamically favorable.
	A SPLET mechanism involving the antioxidant ArOH and the radical ROO^•^ was represented as [121]
	ArOH *⟶* ArO^−^ + H^+^
	ArO^−^ + ROO^•^ *⟶* ArO^•^ + ROO^−^
	ROO^−^ + H^+^ *⟶* ROOH
	or coupling the second and third steps as [122]
TEAC (Trolox Equivalent Antioxidant Capacity) method	ArOH *⟶* ArO^−^ + H^+^
	ArO^−^ + X^•^ + H^+^ *⟶* ArO^•^ + XH
	During the first step the phenolic antioxidant dissociates into its corresponding anion ArO^−^ and a proton, and subsequently the ions which resulted in the first step react with the free radical, yielding a radical form of the phenolic antioxidant ArO^•^ and a neutral molecule XH [122].
	Proton transfer can also occur following electron transfer, as in single electron transfer-proton transfer mechanism (SET-PT) [122]:
	ArOH + X^•^ *⟶* ArOH^•+^ + X^−^
	ArOH^•+^ *⟶* ArO^•^ + H^+^
	During the first step a phenolic antioxidant reacts with the free radical X^•^, yielding a cationic radical ArOH^•+^ derived from the phenolic compound and the anionic form of the radical X^−^. This first step has been reported as thermodynamically significant step. In the second step the cationic radical form of the antioxidant ArOH^•+^ decomposes into a phenolic radical ArO^•^ and a proton [122].

**Table:** (d) Chelation power of antioxidants

Corresponding method	Mechanistic description
Tetramethylmurexide (TMM) assay	Free Cu(II) or Zn(II) which is not complexed by phenolics (e.g., tannins) is bound to tetramethylmurexide (TMM). The complexation with TMM is assessed at 482 nm for Cu(II) and at 462 nm for Zn(II) [123].
Ferrozine assay	Free Fe(II) that is not complexed by phenolics (e.g., tannins) is bound to ferrozine. The complexation of divalent iron with ferrozine is assessed at 562 nm [123].

**Table:** (e) Oxidation of lipids

Corresponding method	Mechanistic description
Peroxide value assessment	Lipid autoxidation results in generation of hydroperoxides, determined iodometrically or colorimetrically [119].
Conjugated diene assay	Fatty acids autoxidation yields conjugated dienes, assessed by UV absorbance at 234 nm [119].
Anisidine assay	Secondary lipid oxidation yields p-anisidine-reactive aldehydes (alkenals, alkadienals, and malondialdehyde), the resulted Schiff base being determined at 350 nm [119].
Thiobarbituric acid reactive substances	Malondialdehyde and unsaturated aldehydes (alkenals and alkadienals) react with thiobarbituric acid; the reaction product is determined photocolorimetrically at 532 nm [119].

**Table 2:** Illustration of the main principles and detection mechanisms in antioxidant activity measurement.

Method for antioxidant capacity assay	Principles underlying the analytical techniques	Detection modes	Ref.
*Chromatographic techniques*			

Thin layer chromatography	The stationary phase is a thin layer of silica gel, aluminium oxide, or cellulose which covers a support of glass, plastic, or aluminium foil. The mobile phase moves by capillarity.	Migration of analytes takes place at different rates due to various repartition coefficients	[91]

High performance thin layer chromatography	It relies on the same principle as conventional TLC but uses a stationary phase with smaller particle size.	Separation performed with improved resolution versus TLC	[93, 94]

Gas chromatography	Separation is based on the repartition between a liquid stationary phase and a gas mobile phase.	Flame ionization, thermal conductivity, or mass spectrometry detection	[124]

Liquid chromatography	Separation is based on the repartition between a solid stationary phase and a liquid mobile. phase	Mass spectrometry or electrochemical detection	[106]

High performance liquid chromatography	Separation is based on the repartition between a solid stationary phase and a liquid mobile phase with distinct polarities at high flow rate and pressure of the mobile phase.	UV-VIS (diode array), fluorescence, mass spectrometry, or electrochemical detection	[108]

*Spectrometric techniques*			

DPPH (2,2-diphenyl-1-picrylhydrazyl) scavenging method	Antioxidant reaction with the nitrogenated radical, followed by absorbance diminution at 515–518 nm.	Photocolorimetry	[125, 126]

TEAC (Trolox Equivalent Antioxidant Capacity) method	Antioxidant reaction with ABTS^*∙*+^ (2,2′-azino-bis(3-ethylbenzothiazoline-6-sulphonic acid cation radical) generated by K_2_S_2_O_8_, followed by blue solution absorbance diminution at 734 nm.	Photocolorimetry	[127]

DMPD (N,N-dimethyl-p-phenylenediamine) method	Reduction of DMPD^*∙*+^ by antioxidants, in the presence of FeCl_3_, with subsequent absorbance decrease at 505 nm.	Photocolorimetry	[128]

FRAP (ferric reducing antioxidant power) method	Reduction of the Fe^3+^-TPTZ (2,4,6-tripyridyl-s-triazine) complex, by sample antioxidants, with absorbance taken at 593 nm.	Photocolorimetry	[129]

PFRAP (potassium ferricyanide reducing power) method	Reduction of potassium ferricyanide by antioxidants, yielding potassium ferrocyanide. The latter reacts with ferric trichloride, and the resulted ferric ferrocyanide blue colored complex is measured at maximum absorbance of 700 nm.	Photocolorimetry	[130]

CUPRAC (cupric reducing antioxidant capacity) method	Cu(II)-neocuproine complex reduction to Cu(I) – bis (neocuproine) chelate, with absorbance recorded at 450 nm.	Photocolorimetry	[131, 132]

Phosphomolybdenum assay	Mo (VI) is reduced Mo (V) by the antioxidants in the sample with generation of a green phosphate/Mo (V) complex at acidic pH, determined at 695 nm.	Photocolorimetry	[133]

Lipid peroxidation activity assay	Antioxidants delay lipid hydroperoxide generation caused by lipoxygenase. The absorbance is measured at 234 nm.	UV absorbance	[106, 134]
	Antioxidants delay radical-induced malonyl dialdehyde generation, as decomposition product of endoperoxides of unsaturated fatty acids, in the presence of thiobarbituric acid. The absorbance is measured at 535 nm.	Photocolorimetry	[106, 135]
	Antioxidants delay conjugated dienes generation as a result of peroxidation of lipid components. The absorbance is measured at 234 nm.	UV absorbance	[85]

Superoxide radical scavenging activity assay	Antioxidants are subject to reaction with a substrate solution containing xanthine sodium salt and 2-(4-iodophenyl)-3-(4-nitrophenol)-5-phenyltetrazolium chloride. Xanthine oxidase is used as biocatalyst and the absorbance increase was monitored at 505 nm.	Photocolorimetry	[136]
	Superoxide anions are generated in a solution containing nitroblue tetrazolium, NADH and phenazine methosulfate. The absorbance taken at 560 nm decreases in the presence of antioxidants, pointing towards superoxide anion scavenging activity.	Photocolorimetry	[137]

Beta carotene bleaching method	Linoleic acid is oxidized by reactive oxygen species. The generated oxidation products such as lipid peroxyl radicals initiate *β*-carotene oxidation and, consequently, its decolorization. Antioxidants delay the discoloration rate, with absorbance measured at 434 nm.	Photocolorimetry	[138, 139]

Xanthine oxidase inhibition assay	Xanthine is used as substrate that yields uric acid as product of XOD-catalyzed reaction. Allopurinol is used as xanthine oxidase inhibitor. Absorbance is measured at 293 nm.	Photocolorimetry	[140]

Superoxide dismutase method	It is assessed in an erythrocyte lysate in the presence of pyrogallol. The enzyme inhibits the autooxidation of the hydroxylated compound, with absorbance read at 420 nm.	Photocolorimetry	[141]
Catalase activity assay	It is measured in an erythrocyte lysate in the presence of H_2_O_2_. The rate of H_2_O_2_ decomposition is assessed at 240 nm.	Photocolorimetry	[142]

Ferrous ion chelating activity assay	Antioxidants react with ferrous salt (e.g., FeCl_2_). Ferrozine as Fe(II) chelator yields a violet complex with absorbance read at 562 nm. The reaction is hindered in the presence of antioxidants that act by chelation, and the result is a decrease of the color of the ferrozine-Fe^2+^ complex, as chelators other than ferrozine act as competing agents for the metal ion.	Photocolorimetry	[143, 144]

ORAC (Oxygen Radical Absorbance Capacity) assay	Antioxidants scavenge the peroxyl radicals, induced by 2,2′-azobis-(2-amidino-propane) dihydrochloride (AAPH) decomposition, slowing the fluorescent decay of fluorescein or phycoerythrin.	Fluorimetry	[145–147]

HORAC (Hydroxyl Radical Antioxidant Capacity) assay	Antioxidants quench OH radicals formed in a Fenton-like system.	Fluorimetry	[148]

TRAP (Total Radical Trapping Antioxidant Parameter) assay	The rate of peroxyl radical generation by 2,2′-diazobis-2-amidinopropane dihydrochloride (ABAP) is quantified through the fluorescence diminution of the protein R-phycoerythrin.	Fluorescence	[149, 150]

Horseradish peroxidase-luminol-hydrogen peroxide chemiluminescent assay	Horseradish peroxidase catalyses luminol oxidation by H_2_O_2_ with light emission. Light emission is quenched by antioxidants.	Chemiluminescence	[151]

*Electrochemical techniques*			

Cyclic voltammetry	The potential is linearly swept in a triangular waveform.	The analytical signal is represented by the intensity of the cathodic/anodic peak	[152, 153]

Differential pulse voltammetry	Potential voltage pulses are superimposed on the potential scan, which is performed linearly or stairstep-wise.	First current sampling before applying the pulse and the second towards the end of the pulse period	[154, 155]

Square-wave voltammetry	A square wave is superimposed on the potential staircase sweep.	Current intensity recorded at the end of each potential change	[155, 156]

Amperometry	The potential of the working electrode is maintained at a constant value versus the reference electrode.	Current intensity generated by the oxidation/reduction of an electroactive analyte	[157]

Biamperometry	The reaction of the antioxidant with the oxidized form of a reversible indicating redox couple in an electrochemical cell containing two identical electrodes.	The current flowing between two identical working electrodes at a constant small applied potential difference	[158–160]

Potentiometry	The analytical signal represented by the potential change is the result of the variation of an ionic species concentration. The antioxidants react with the oxidized form of a redox couple, altering the concentration ratio between the oxidized form and the reduced form.	Potential change after reaction of antioxidants with an indicating redox couple	[161]

**Table 3:** Significant examples of total antioxidant capacity assessment in plants.

Number	Analysed products (extracts)	Compounds determined	Applied analytical technique	Health benefits as they appear in the cited studies	Ref.
(1)	Leaves from cherry tree, peach tree, plum tree, olive tree, pear tree, apple tree, pistachio, and chestnut	(i) Total phenols (ii) Nonflavonoids phenol (iii) Total antioxidant capacity	(i) DPPH assay (ii) FRAP assay	Used in pharmaceutical purposes and also act as natural pesticides and beverage ingredients	[162]

(2)	Leaf extracts from six *Vitis vinifera* L. varieties	(i) Total phenols (ii) Flavonoids, nonflavonoids, and flavanols (iii) Total antioxidant capacity	(i) HPLC (ii) DPPH assay (iii) FRAP assay	Antimicrobial activity	[163]

(3)	Tropical herbs: *Momordica charantia*, *Centella asiatica*, and *Morinda citrifolia*	(i) Catechin (ii) Total antioxidant capacity	(i) HPLC (ii) DPPH assay (iii) FRAP assay	Inhibitors of pancreatic lipase activity	[164]

(4)	Edible and medicinal *Acacia albida* organs (leaves and bark)	(i) Polyphenols (ii) Total antioxidant capacity	(i) HPLC (ii) DPPH assay (iii) ABTS assay	Traditionally used to treat colds, flu, fever, tooth decay, vomiting, diarrhea, urinary disorders, malaria, and inflammation	[165]

(5)	*Citrus* fruits	Total antioxidant capacity	(i) HPLC free radical scavenging detection (ii) DPPH assay (iii) ABTS assay		[166]

(6)	*Salvia* sp. and *Plantago* sp.	(i) Total phenolic content (ii) Total antioxidant capacity	(i) UV-Vis fingerprint (ii) DPPH assay	Helpful in preventing different diseases	[167]

(7)	*Ajuga iva* (leaf extracts)	(i) Total phenolic content (ii) Total flavonoids (iii) Total antioxidant capacity	(i) DPPH assay (ii) FRAP assay	Diuretic, cardiac tonic, and hypoglycemic	[168]

(8)	*Filipendula vulgaris*	(i) Total phenolic content (ii) Total antioxidant capacity	(i) DPPH assay (ii) ABTS assay	(i) Antibacterial activity (ii) Fights against inflammatory diseases, rheumatoid arthritis, and gout	[169]

(9)	*Asphodelus aestivus* Brot.	Total antioxidant capacity	(i) FRAP assay (ii) DPPH assay (iii) ABTS assay	(i) Are used against hemorrhoids, nephritis, burns, and wounds (ii) Gastroprotective effect against ethanol-induced lesions	[170]

(10)	*Melia azedarach* (Chinaberry) (bark extract)	Total antioxidant capacity	DPPH assay	Antimicrobial agents in various infectious diseases	[171]

(11)	Bitter bean, *Parkia speciosa*	(i) Total phenolic constituents (ii) Total antioxidant capacity	(i) HPLC (ii) Folin-Ciocalteu method (iii) DPPH assay (iv) ABTS assay	(i) Antibacterial effects on kidney, ureter, and urinary bladder (ii) Diuretic and relaxing properties (iii) Seed extracts were reported to possess hypoglycemic, anticancer, and antiangiogenic activities	[172]

(12)	*Brassica oleracea* L.	(i) Glucosinolates (ii) Total phenolic constituents (iii) Ascorbic acid (iv) Total antioxidant capacity	(i) HPLC (ii) Folin-Ciocalteu method (iii) DPPH assay	(i) Neutralizes carcinogens (ii) Attenuates cancer cell division (iii) Accelerates the atrophy of cancer cells with damaged DNA	[116]

(13)	Grape pomace seed and skin extracts	(i) Total phenols (ii) Total anthocyanins (iii) Total tannins (iv) Total antioxidant capacity	(i) HPLC MS (ii) DPPH assay (iii) TEAC assay (iv) ABTS assay (v) Folin-Ciocalteu method	Limit the oxidation of nucleic acids, proteins, and lipids, which may initiate degenerative diseases	[173]

(14)	*Diplotaxis simplex* (Brassicaceae) (flower, leaf, and stem extracts)	(i) Total phenols, flavonoids, and proanthocyanidins (ii) Total antioxidant capacity	ORAC assay	Anti-inflammatory activity	[174]

(15)	Cereal grains (24 cereal grains from China)	(i) Total phenolic constituents (ii) Total antioxidant capacity	(i) FRAP assay (ii) TEAC assay (iii) HPLC (iv) Folin-Ciocalteu method	Reduces the risk of cardiovascular diseases and reduces type II diabetes, ischemic stroke, and some cancers	[105]

(16)	Some cereals and legumes	(i) Total phenolic constituents (ii) Total antioxidant capacity	(i) Folin-Ciocalteu method (ii) DPPH assay (iii) FRAP assay	(i) Reduces the incidence of age-related chronic diseases (ii) Reduces heart diseases and some types of cancer	[175]

(17)	*Clusia fluminensis* Planch. & Triana	(i) Flavonoids content (ii) Total antioxidant capacity	(i) Photometric assay based on aluminum chloride complex formation (ii) DPPH assay	(i) Antifungicidal activity (ii) Protection against cardiovascular diseases	[176]

(18)	Bitter cumin (*Cuminum nigrum* L.)	(i) Total phenolic constituents (ii) Total antioxidant capacity	(i) HPLC (ii) DPPH assay	(i) Antibacterial activity (ii) Reduces risk of cancer and cardiovascular diseases	[106]

(19)	Essential oils of *Cynanchum chinense* and *Ligustrum compactum*	Total antioxidant capacity	(i) DPPH assay (ii) ABTS assay	(i) Anticonvulsant (ii) Antitumor (iii) Antimicrobial	[177]

(20)	*Caspicum annum* L. grossum sendt.; *Rosmarinus officinalis*	(i) Total phenolic constituents (ii) Total antioxidant capacity	(i) Folin-Ciocalteu method (ii) ABTS assay		[178]

(21)	*Diospyros bipindensis* (Gürke)	(i) Plumbagin, canaliculatin, ismailin, betulinic acid, and 4-hydroxy-5-methyl-coumarin (ii) Total antioxidant capacity	(i) HPLC, NMR, and MS analyses (ii) DPPH assay (iii) ABTS assay (iv) ORAC assay	Anti-inflammatory and antimicrobial activities	[179]

(22)	*Carissa opaca* fruits	Total flavonoids content	HPLC	(i) Antibacterial activity (ii) Anticancer activity (iii) Antitumoral activity	[102]

(23)	*Artemisia capillaris herba*	(i) Total phenolic constituents (ii) Total antioxidant capacity	(i) HPLC MS (ii) DPPH assay (iii) *β*-carotene bleaching method	(i) Cholagogic, antipyretic, anti-inflammatory, and diuretic in jaundice (ii) Used against inflammation of the liver and cholecyst	[114]

(24)	*Lantana camara* (various parts: leaf, root, fruit, and flower)	(i) Total phenolic constituents (ii) Total antioxidant capacity	(i) DPPH assay (ii) Folin-Ciocalteu method	Used against itches, cuts, ulcers, rheumatism, eczema, malaria, tetanus, and bilious fever	[180]

(25)	Grape extracts	(i) Total phenolic constituents (ii) Total anthocyanins (iii) Tannins (iv) Total antioxidant capacity	(i) Folin-Ciocalteu method (ii) Binding with polyvinylpyrrolidone (iii) ABTS assay		[181]

(26)	*Scutellaria baicalensis* radix	Total antioxidant capacity	DPPH assay	Used in hepatitis and inflammation of the respiratory and gastrointestinal tract	[182]

(27)	*Lycium* species	(i) Total phenolic constituents (ii) Total antioxidant capacity	(i) HPLC (ii) DPPH assay	Diuretic, antipyretic, tonic, aphrodisiac, hypnotic, hepatoprotective, and emmenagogic	[107]

(28)	Dried fruits consumed in Algeria (prunes, apricots, figs, and raisins)	(i) Total phenolic constituents (ii) Total anthocyanins (iii) Total antioxidant capacity	(i) Folin-Ciocalteu method (ii) DPPH assay (iii) Phosphomolybdenum method	Reduce the risk of cancer and heart disease	[183]

(29)	*Rubus grandifolius* Lowe (leaves, flowers, and berries)	(i) Total antioxidant capacity (ii) Total phenolic constituents	(i) DPPH assay (i) ABTS assay (iii) FRAP assay (iv) HPLC	Acts as astringent and as remedy for diabetes and is depurative and diuretic and relieves sore throat	[184]

(30)	Red pitaya (*Hylocereus polyrhizus*) seed	(i) Total antioxidant capacity (ii) Total phenolic constituents (iii) Flavonoids content	(i) DPPH assay (ii) Folin-Ciocalteu method (iii) HPLC		[185]

(31)	Cornelian cherry, Japanese persimmon, and cherry laurel	(i) Total phenolic content (ii) Total flavonoids content (iii) Total antioxidant capacity	(i) Folin-Ciocalteu method (ii) DPPH assay (iii) FRAP assay (iv) CUPRAC assay	Able to provide prevention of diseases	[186]

(32)	*Inula crithmoides* L.	(i) Total phenolic content (ii) Total antioxidant capacity	(i) Folin-Ciocalteu method (ii) DPPH assay	Antibacterial, antifungal, and cytotoxic	[187]

(33)	*Lycium intricatum* Boiss.	(i) Total phenolic content (ii) Total antioxidant capacity	(i) Folin-Ciocalteu method (ii) HPLC (iii) DPPH assay (iv) ABTS assay (v) FRAP assay	Decreases the risk of diseases such as cancer, neurodegenerative disorders, and cardiovascular diseases	[188]

(34)	*Millingtonia hortensis* Linn. parts (leaves, stem, root, and flower)	(i) Total phenolic content (ii) Total antioxidant capacity	(i) Folin-Ciocalteu method (ii) DPPH assay	Reduces risks of diabetes, cancer, and cardiovascular diseases	[189]

(35)	*Ononis natrix*	(i) Total phenolic content (ii) Total antioxidant capacity	(i) Folin-Ciocalteu method (ii) DPPH assay	Antimicrobial activities	[190]

(36)	*Citrus grandis* Osbeck	Total antioxidant capacity	DPPH assay		[191]

(37)	*Sorbus torminalis* (L.) Crantz (wild service tree) fruits	(i) Total phenolic content (ii) Total flavonoids content (iii) Total antioxidant capacity	(i) Folin-Ciocalteu method (ii) ABTS assay (iii) DPPH assay	Used in treatment of cardiac diseases and Alzheimer's disease	[192]

(38)	*Rosmarinus officinalis*	(i) Total phenolic content (ii) Total antioxidant capacity	(i) HPLC (ii) DPPH assay (iii) TEAC assay		[104]

(39)	*Sapindus mukorossi* Gaertn.	(i) Total phenolic content (ii) Total antioxidant capacity	(i) Folin-Ciocalteu method (ii) DPPH assay	Fights against heart disease, aging, diabetes mellitus, and cancer	[193]

(40)	11 medicinal Algerian plants	(i) Total phenolic content (ii) Total antioxidant capacity	(i) Folin-Ciocalteu method (ii) HPLC (iii) ABTS assay (iv) TEAC assay	Antitumoral, anticancer, analgesic, diuretic, analgesic, and so forth	[103]

(41)	Six *Teucrium arduini* L. populations	(i) Total phenolic content (ii) Total antioxidant capacity	(i) Folin-Ciocalteu method (ii) FRAP assay (iii) ABTS assay (iv) DPPH assay	Hypoglycemic, antipyretic, antiulcerative, and antibacterial	[194]

(42)	*Vitex agnus-castus* (*Vitex AC*)	Total antioxidant capacity	(i) ABTS assay (ii) DPPH assay (iii) FRAP assay (iv) CUPRAC assay	Cytotoxic activities against various types of cancer cells	[195]

(43)	*Andrographis paniculata*	(i) Total antioxidant capacity (ii) Total phenolic content (iii) Total andrographolides concentration	(i) DPPH assay (ii) FRAP assay (iii) CUPRAC assay (iv) HPLC-DAD (v) LC-MS/MS (vi) GC-MS	(i) Treats dyspepsia, influenza, dysentery, malaria and respiratory infections (ii) Antidote for snakebites and poisonous stings (iii) Active in cytotoxicity tests against cancer cell lines	[111]

(44)	*Hypericum perforatum* L., *Matricaria recutita* L., *Achillea millefolium* L., *Thymus vulgaris* L., and *Salvia officinalis* L.	(i) Total antioxidant capacity (ii) Total phenolic content	(i) Thin layer chromatography (ii) LC MS (iii) DPPH assay	Anti-inflammatory, antiviral, antimicrobial, antiallergic, anticancer, antiulcer, and antidiarrheal	[91]

(45)	*Celastrus paniculatus* Willd.	Total antioxidant capacity	(i) DPPH assay (ii) FRAP assay (iii) TEAC assay (iv) GC MS	Calmant	[196]

(46)	Cerrado Brazilian fruits	(i) Total phenolic content (ii) Total antioxidant capacity	(i) Folin-Ciocalteu method (ii) ABTS assay	Chemopreventive effects	[197]

(47)	Buckwheat (*Fagopyrum esculentum* Moench)	(i) Total phenolic content (ii) Total antioxidant capacity	(i) HPLC (ii) DPPH assay		[198]

(48)	Green and black tea infusions, herbal infusions, and fresh fruit extracts	Total antioxidant capacity	Potentiometric and flow injection		[161]

(49)	Cocoa beans (raw, preroasted, and roasted)	(i) Total phenolic content (ii) Total antioxidant capacity	(i) Folin-Ciocalteu method (ii) DPPH assay (iii) ABTS assay		[199]

(50)	Rapeseed and its products	(i) Total phenolic content (ii) Total antioxidant capacity	(i) Silver nanoparticle-based method (ii) Folin-Ciocalteu method (iii) DPPH assay (iv) FRAP assay		[200]

(51)	Edible plants (broccoli, cauliflower, strawberry, tomato, potato, and corn)	Total antioxidant capacity	Cyclic voltammetry		[201]

(52)	Herb extracts from the Labiatae family	Total antioxidant capacity	(i) DPPH assay (ii) Amperometric	Antioxidant in food industry	[202]

(53)	Indian mushrooms (*Agaricus bisporus*, *Hypsizygus ulmarius*, and *Calocybe indica*)	(i) Total phenolic content (ii) Total antioxidant capacity	(i) DPPH assay (ii) FRAP assay (iii) Folin-Ciocalteu method (iv) Cyclic voltammetry	Provides health benefits and protection against degenerative diseases	[203]

(54)	Three types of algae: *Spirulina subsalsa* and *Selenastrum capricornutum* (both cultivated) and (powdered) *Spirulina maxima*	Total antioxidant capacity	(i) Amperometric using the enzymatic biosensor with superoxide dismutase (ii) Cyclic voltammetry	Antiaging potential	[204]

(55)	Buckwheat sprouts (roots obtained from dark- and light-grown)	Total antioxidant capacity	(i) TEAC assay (ii) Cyclic voltammetry		[205]

(56)	Tea infusions	(i) Total phenolic content (ii) Total antioxidant capacity	(i) HPLC (ii) Cyclic voltammetry	Reduce blood glucose level	[206]

(57)	*Coriandrum sativum*	Antioxidant terpenes	HPTLC	digestive, anti-inflammatory, antimicrobial, hypolipidemic, antimutagenic, and anticarcinogenic	[96]

(58)	*Scoparia dulcis*	Flavonoids and terpenoids	HPTLC	Antibacterial, antifungal, antiherpetic, anti-inflammatory, antiseptic, antispasmodic, antiviral, cytotoxic, emmenagogic, emollient, febrifuge, and hypotensive	[95]

(59)	*Acacia confusa*	(i) Total phenolic content (ii) Total antioxidant capacity	(i) Folin-Ciocalteu method (ii) DPPH assay	Used for wound healing and antiblood stasis	[207]

(60)	Teas and herbal infusions	(i) Total phenolic content (ii) Total antioxidant capacity	(i) Folin-Ciocalteu method (ii) DPPH assay (iii) FRAP assay (iv) ABTS assay (v) Polarographic		[208]

(61)	Extra virgin oils	Total phenolic content	Voltammetric		[209]

(62)	Selected wines	(i) Total phenolic content (ii) Total antioxidant capacity	(i) Folin-Ciocalteu method (ii) DPPH assay (iii) Differential pulse voltammetry		[210]

(63)	Fruits (raspberry, strawberry, and berry fruit) and vegetables (carrot, tomato, and rhubarb)	Antioxidant capacity	Differential pulse voltammetry		[211]
